# Comprehensive Anaemia Programme and Personalized Therapies (CAPPT): protocol for a cluster-randomised controlled trial testing the effect women’s groups, home counselling and iron supplementation on haemoglobin in pregnancy in southern Nepal

**DOI:** 10.1186/s13063-022-06043-z

**Published:** 2022-03-01

**Authors:** Naomi M. Saville, Chandani Kharel, Joanna Morrison, Helen Harris-Fry, Philip James, Andrew Copas, Santosh Giri, Abriti Arjyal, B. James Beard, Hassan Haghparast-Bidgoli, Jolene Skordis, Adam Richter, Sushil Baral, Sara Hillman

**Affiliations:** 1 Institute for Global Health, University College London (UCL), London, UK; 2 HERD International, Thapathali, Kathmandu, Nepal; 3 Department of Population Health, London School of Hygiene & Tropical Medicine (LSHTM), London, UK; 4 Guildford, GU1 3LD UK; 5 Vagelos College of Physicians and Surgeons, Columbia University, New York, NY USA; 6 Health Research and Social Development Forum (HERD), Kathmandu, Nepal; 7 Institute for Women’s Health, University College London (UCL), London, UK

**Keywords:** Anaemia, Pregnant woman, Menstrual monitoring, Home visiting, Haemoglobin, Participatory Learning and Action, Community-intervention, Cluster randomised controlled trial, Nepal

## Abstract

**Background:**

Anaemia in pregnancy remains prevalent in Nepal and causes severe adverse health outcomes.

**Methods:**

This non-blinded cluster-randomised controlled trial in the plains of Nepal has two study arms: (1) Control: routine antenatal care (ANC); (2) Home visiting, iron supplementation, Participatory Learning and Action (PLA) groups, plus routine ANC. Participants, including women in 54 non-contiguous clusters (mean 2582; range 1299–4865 population) in Southern Kapilbastu district, are eligible if they consent to menstrual monitoring, are resident, married, aged 13–49 years and able to respond to questions. After 1–2 missed menses and a positive pregnancy test, consenting women < 20 weeks’ gestation, who plan to reside locally for most of the pregnancy, enrol into trial follow-up. Interventions comprise two home-counselling visits (at 12–21 and 22–26 weeks’ gestation) with iron folic acid (IFA) supplement dosage tailored to women’s haemoglobin concentration, plus monthly PLA women’s group meetings using a dialogical problem-solving approach to engage pregnant women and their families. Home visits and PLA meetings will be facilitated by auxiliary nurse midwives. The hypothesis is as follows: Haemoglobin of women at 30 ± 2 weeks’ gestation is ≥ 0.4 g/dL higher in the intervention arm than in the control. A sample of 842 women (421 per arm, average 15.6 per cluster) will provide 88% power, assuming SD 1.2, ICC 0.09 and CV of cluster size 0.27.

Outcomes are captured at 30 ± 2 weeks gestation. Primary outcome is haemoglobin concentration (g/dL). Secondary outcomes are as follows: anaemia prevalence (%), mid-upper arm circumference (cm), mean probability of micronutrient adequacy (MPA) and number of ANC visits at a health facility. Indicators to assess pathways to impact include number of IFA tablets consumed during pregnancy, intake of energy (kcal/day) and dietary iron (mg/day), a score of bioavailability-enhancing behaviours and recall of one nutrition knowledge indicator. Costs and cost-effectiveness of the intervention will be estimated from a provider perspective. Using constrained randomisation, we allocated clusters to study arms, ensuring similarity with respect to cluster size, ethnicity, religion and distance to a health facility. Analysis is by intention-to-treat at the individual level, using mixed-effects regression.

**Discussion:**

Findings will inform Nepal government policy on approaches to increase adherence to IFA, improve diets and reduce anaemia in pregnancy.

**Trial registration:**

ISRCTN 12272130.

**Supplementary Information:**

The online version contains supplementary material available at 10.1186/s13063-022-06043-z.

## Administrative information

Note: the numbers in curly brackets in this protocol refer to SPIRIT checklist item numbers. The order of the items has been modified to group similar items (see http://www.equator-network.org/reporting-guidelines/spirit-2013-statement-defining-standard-protocol-items-for-clinical-trials/).

**Table:** 

Title {1}	Comprehensive Anaemia Programme and Personalized Therapies (CAPPT): Protocol for a cluster-randomised controlled trial testing the effect women’s groups, home counselling and iron supplementation on haemoglobin in pregnancy in southern Nepal
Trial registration {2a and 2b}.	ISRCTN registration number: 12272130. Trial Registry name: ISRCTN Date of registration: 22 April 2021. Trial registry URL: 10.1186/ISRCTN12272130 ISRCTN collects all items from the World Health Organization Trial Registration Data Set.
Protocol version {3}	Version number 1.4, 15 Jan 2022
Funding {4}	UK Medical Research Council (MRC) / Newton Fund (MR/R020485/1).
Author details {5a}	Naomi M. Saville^1*^, Chandani Kharel^2^, Joanna Morrison^1^, Helen Harris-Fry^3^, Philip James^3^, Andrew Copas^1^, Santosh Giri^2^, Abriti Arjyal^2^, B. James Beard^4^, Hassan Haghparast-Bidgoli^1^, Jolene Skordis^1^ Adam Richter^5^, Sushil Baral^2,6^, Sara Hillman^7^ 1. Institute for Global Health, University College London (UCL), London, UK 2. HERD International, Thapathali, Kathmandu, Nepal 3. Department of Population Health, London School of Hygiene & Tropical Medicine (LSHTM), London, UK 4. Independent consultant, Guildford, GU1 3LD, UK. 5. Vagelos College of Physicians and Surgeons, Columbia University, New York, NY, USA 6. Health Research and Social Development Forum (HERD), Kathmandu, Nepal 7. Institute for Women’s Health, University College London (UCL), London, UK
Name and contact information for the trial sponsor {5b}	Health Research and Social Development Forum (HERD) Nepal, PO Box 24133, Prasuti Griha Marg, Thapathali, Kathmandu, Nepal.
Role of sponsor {5c}	The trial sponsor HERD Nepal under the leadership of Sushil Baral, working with the trial coordinating centre, University College London Institute for Women’s Health, will take responsibility for all aspects of study design; collection, management, analysis, and interpretation of data; writing of the report; and the decision to submit the report for publication. The ultimate authority over these activities is shared between HERD and UCL. The funders have no role in study design; collection, management, analysis, and interpretation of data; writing of the report; or the decision to submit the report for publication.

## Introduction

### Background and rationale {6a}

Anaemia in pregnancy is associated with low birth weight [1–3], perinatal mortality [4] and maternal mortality, with 18% of maternal deaths attributable to severe anaemia [5]. A 1 g/dL increase in haemoglobin in late pregnancy can reduce the risk of maternal mortality by 20% [6] cited in [7]. Despite government provision of free iron/folic acid (IFA) to pregnant women in many low- and middle-income countries (LMICs), anaemia levels in pregnancy remain alarmingly high [8, 9]. The global burden of anaemia in pregnancy is estimated to be as high as 40% [10] and 90% of severe cases are in LMICs, particularly in Africa and South Asia [4, 9, 11, 12].

The aetiology of anaemia is multifactorial [13–15]. Dietary iron deficiency is the most common cause of anaemia [12, 13] but other micronutrient deficiencies [1, 16, 17], inherited blood disorders or haemoglobinopathies (sickle cell anaemia and thalassaemia) [18] and parasitic infestations (e.g. malaria and hookworm) [19] are also responsible [12, 15]. Iron deficiency anaemia is estimated to affect 25% [13] to > 50% [15, 20] of individuals globally.

In Nepal, estimates of anaemia in pregnant women (PW) vary from 27% according to the 2016 Nepal National Micronutrient Status Survey (NNMSS) [21], to 46% according to the 2016 Nepal DHS survey [22]. The burden of anaemia is highest at 52% in the *Terai* (plains) compared to the hills and mountains and varies by season [23], ethnic group, and is higher amongst adolescents, farmers, women of short stature and in women married to illiterate men [24].

The extent to which iron deficiency is driving anaemia in Nepal is difficult to ascertain. The NNMSS found iron deficiency anaemia (IDA) in only 5% of PW [21], but a global review attributed iron deficiency to 55% of the anaemia burden in South Asia [12]. Other micronutrient deficiencies in Nepal may also contribute to anaemia [1, 17, 25], although these prevalence estimates also vary widely, at 3–7% for vitamin A, 28–42% for B12, 12–90% for folate, and 24–90 % for zinc [21, 26–28]. This varied and discordant evidence around iron deficiency and anaemia in pregnancy supports the development of a multi-pronged intervention that keeps iron deficiency central but also targets general enhancement of diet and health in pregnancy.

#### Insufficient dietary intake

Micronutrient deficiencies in Nepalese women are largely attributable to inadequate diets. Although dietary inadequacies have been reported for many micronutrients [29–32], attaining adequate dietary iron intake is particularly challenging [29, 30, 33, 34], especially in pregnancy due to the increased requirements. In the plains of Nepal, the probability of dietary iron adequacy is only 20% and intake of animal-source foods are low [30]. Dietary intake is also inequitable, with gender-based discrimination and food restrictions preventing PW from accessing high status, relatively expensive micronutrient-rich foods [31, 35–38]. Low awareness of dietary needs in pregnancy [36], food taboos [38] and household power hierarchies [36] may also prevent households from providing micronutrient-rich foods to PW even when they are available. Women may also ‘eat down’ or eat less than they did before pregnancy for numerous reasons, including fear of obstructed labour if the baby is large [39], religious fasting, misconceptions about the stomach/intestines ‘squashing’ the baby, morning sickness (especially in early pregnancy), discomfort or indigestion from eating large meals [40], food aversion and proscriptions such as avoiding foods believed to heat the body [41, 42].

#### IFA supplementation and antenatal care

A Cochrane review of 44 randomised controlled trials found that iron supplementation during pregnancy reduced the risk of maternal anaemia at term by 70% [43]. Accordingly, IFA supplementation in pregnancy has been implemented in many LMICs, including Nepal since 1997. IFA is an integral part of the Government of Nepal (GoN)’s Safe Motherhood programme which also recommends at least four antenatal care (ANC) visits at health facilities at 4, 6, 8 and 9 months of gestation, though recently four additional antenatal ‘contacts’ with a skilled provider are also being recommended. PW are eligible to receive free IFA from 14 weeks gestation for 180 days of pregnancy and 45 days post-partum from health workers or female community health volunteers (FCHVs). If a PW is anaemic (< 11.0 g/dL), GoN recommends the daily IFA dose is doubled from 60 to 120 mg/day (per current WHO guidance [44]), though this is not routinely practised. All PW are also advised to take a single dose of 400 mg of albendazole after the first trimester to reduce the risk of anaemia from hookworm infection [45].

In 2016, Nepal was able to provide some IFA supplementation to 91% of PW, but only 42% completed the minimum 180-day IFA dose. Increasing adherence to IFA could reduce anaemia; a study from the *Terai* found higher odds of anaemia amongst women who took lower doses of IFA (≤ 143 vs ≥ 144 tablets) [46]. To maximise IFA intake, ANC needs to begin earlier in pregnancy [47]. In 2016, 31% of PW in Nepal had fewer than 4 ANC visits and 42% of rural women had their first ANC after 4 months [22]. In peri-urban breastfeeding Nepalese woman, anaemia appears to have been prevented through IFA supplementation. Women’s dietary iron adequacy was 30% and bioavailability of non-haem iron was low due to high levels of phytates in the diet, yet only 5% reported low plasma ferritin iron deficiency anaemia, suggesting that IFA had filled the gap [33]. In many places however, supply-side issues may still prevent women from receiving the necessary dose free of cost and some may discontinue IFA consumption due to side effects [47, 48].

Knowledge regarding anaemia, iron deficiency, and IFA supplementation remains low amongst PW, and improved social support to minimise barriers to uptake and better understanding of the severe implications of anaemia have been suggested as means of improving IFA compliance [47, 49]. ANC counselling should discuss benefits of taking IFA, how to manage side effects, how to increase intake and bioavailability of dietary iron and signs of anaemia [44], but time is short for high-quality counselling in busy health facilities.

#### Other drivers of anaemia in Nepal: infections, inflammation and haemoglobinopathies

In addition to inadequate diets and IFA intakes, infections and inflammation are important in the aetiology of anaemia [15, 21]. Hookworm infestations are prevalent in Nepalese PW [50, 51], and anaemia prevalence was higher amongst PW who had not consumed deworming medication in the past 6 months compared to those who had [21], highlighting the importance of quality ANC to detect and treat infections.

Iron metabolism is influenced by haemoglobinopathies [15, 52] and by genetic variation in individuals’ ability to absorb iron [53]. The NNMSS tested NPW for blood disorders and found 1% had alpha-thalassemia, 3% beta-thalassemia, 1% sickle cell and 14% glucose-6-phosphate dehydrogenase deficiency [21]. These haemoglobinopathies, which account for 12% of female anaemias worldwide [11] and are associated with low haemoglobin during pregnancy [54], are usually not amenable to iron treatment and may limit the effectiveness of IFA supplementation [15] in a small proportion of cases.

A combined approach of improving diets, increasing IFA uptake and tailored dosage according to guidelines, and reducing infection could be an efficient way of tackling anaemia in Nepal, especially in *Terai* populations where anaemia prevalence is high.

#### Potential interventions to improve anaemia in Nepal

With IFA supplementation routinely provided in many LMICs yet anaemia remaining stubbornly prevalent, effective behaviour change interventions in pregnancy are needed to increase IFA compliance, enhance dietary micronutrient intake and bioavailability, and reduce infections/inflammation through deworming [44].

Home visiting counselling and nutrition education approaches have shown promise. In India, a home visiting nutritional counselling model for PW reduced anaemia from 96 to 79% and improved Hb by > 1 g/dL [55]. In Nepal, an education programme with routine iron supplementation improved haemoglobin levels in PW by up to 0.26 g/dL and reduced anaemia prevalence by 65% [56]. However, a systematic review of nutrition education and counselling (NEC) interventions found that effects are highly variable. When combined with provision of food or supplements, women’s risk of anaemia in late pregnancy was reduced by 42%, but without this NEC effects were smaller (16% lower risk) and only marginally significant [57]. In situations or environments that are not enabling, educating women and families may not automatically provoke changes in behaviour. A long literature on behaviour change theory [58–60] and qualitative research [36] indicates that nutrition education may need to be coupled with additional components that address wider contextual factors that enable women and families to implement new knowledge. As indicated by the NEC review, this includes access to food and supplements. Other studies, including our formative research, suggest this also includes addressing complex factors such as gender norms, power hierarchies, community cohesion and trust in health services [61].

To address some of these wider community-level factors, women’s groups may be an effective intervention, but as with NEC, a review of 36 studies on the effects of women’s groups found highly heterogeneous effects on nutrient intakes [62]. This heterogeneity may be due to differences in implementation, context, or ways the intervention interacts with context. Researchers have suggested a typology of women’s groups to help identify differences in approach: classrooms (didactic behaviour change), clubs (build relationships between members), and collectives (engage the whole community) [63]. In the case of anaemia, a ‘collective’ approach may be needed. One form of collective approach uses Participatory Learning and Action (PLA), which follows a four-phase ‘cycle’ of problem identification, planning strategies to overcome the problems, implementing the strategies and evaluating them. The PLA approach is based on the theory that many health problems are rooted in powerlessness and may be addressed by social and political empowerment [64–66]. Hypothesised pathways to impact include women sharing experiences and motivating each other to try new behaviours, collective ownership of nutrition problems, increasing resources to afford better nutrition and changing social norms to promote healthy behaviours. Several interventions using PLA groups in South Asia have been highly effective at improving health outcomes, particularly reducing maternal and neonatal mortality [67–73] and diabetes [74]. However, effects on women’s diets and anthropometry have been less consistent, showing small [75], mixed [76–78] and sometimes null effects [74]. The only study that has reported effects of PLA on haemoglobin is the UPAVAN trial in India which combined PLA with an agricultural intervention [79], which showed no impact on mothers’ haemoglobin or MUAC but did improve dietary diversity [80].

#### CAPPT rationale

Taken together, evidence demonstrates that anaemia could be reduced in rural Nepal by (1) increasing adherence to WHO recommendations on IFA (tailoring dose according to anaemia status and increasing PW’s compliance), (2) improving diets to increase intakes of iron, bioavailability of iron and other micronutrients, and (3) increasing access to deworming tablets. Previous trials suggest that both PLA and NEC could improve diets and haemoglobin concentrations, but evidence of their effectiveness when integrated has not been studied. Hence, CAPPT will test an intervention to reduce anaemia by addressing IFA, diets and deworming, using a combined approach of PLA groups with two nutrition counselling home visits to PW at home in a disadvantaged population in the Nepal *Terai*.

The CARING trial in India showed that a single home visit to third trimester pregnant women, combined with PLA groups, improved PW’s dietary diversity but not MUAC [76], so our model of two home-based counselling visits in early to mid-pregnancy combined with PLA groups could also improve diets. Experience from the Low Birth Weight South Asia Trial (LBWSAT) in the *Terai* showed that PLA groups wanted to implement home visits as part of their strategies [77]; women may be unable to leave the home in pregnancy, especially during their first pregnancy [81]; and PLA group attendance by pregnant women was a higher when PLA was combined with cash or food transfers [77]. Home visits might work synergistically to encourage PLA group attendance, reach women who cannot/do not leave their homes and engage household members who oversee food purchasing and allocation decisions (males and mothers-in-law) [36]. We hypothesise that our planned home visiting intervention will facilitate personalised, direct one-to-one support to PW and their families, whilst the PLA groups will work at the community level to create an enabling environment, changing community-level norms, and facilitate shared exchange of nutrition knowledge and peer support amongst group members.

### Objectives {7}

The primary objective of the Comprehensive Anaemia Programme and Personalized Therapies (CAPPT) trial is to assess the impact on haemoglobin (Hb) at 30 ± 2 weeks of pregnancy, of an integrated intervention providing personalised nutrition counselling at pregnant women’s homes, together with tailored dosage of oral iron-folic acid (IFA) and PLA women’s groups in the community, in addition to routine ANC, compared with a control arm where women have access to routine ANC only.

Secondary objectives are as follows:

1. Assess the impact at 30 ± 2 weeks gestation of this integrated intervention by comparing prevalence of anaemia, mean probability of adequacy (MPA) of 11 micronutrients, mid-upper arm circumference (MUAC) between study arms and count of ANC visits at a health facility.
2. Explore potential pathways to impact by comparing between study arms: count of IFA supplements consumed; daily energy and iron intakes, behaviours to enhance bioavailability, nutrition knowledge.
3. Compare intervention effects between population subgroups such as wealth groups, baseline BMI category and anaemia levels.
4. Undertake a dose-response analysis to analyse the effect of different levels of exposure to PLA groups, home visits and number of IFA consumed, separately and in combination
5. Conduct a process evaluation to describe exposure, implementation and fidelity of the intervention to that planned and measure hypothesised changes in target behaviours of pregnant women and their families including bargaining power and decision-making power, equity of food and nutrient allocation between PW and their husbands, experience of side effects of iron therapy and health literacy.
6. Estimate cost and cost-effectiveness of the intervention package from a provider perspective.

### Trial design {8}

This is a non-blinded parallel group two-arm cluster-randomised controlled trial, with an allocation ratio of 1:1, conducted in Kapilbastu district in the rural plains of Nepal. Trial arms are as follows: (1) control (routine antenatal care (ANC); (2) ‘Home visiting plus PLA’ intervention package comprising a combination of tailored IFA supplementation and counselling at home, and Participatory Learning and Action (PLA) meetings held in the community, in addition to routine ANC.

Our study protocol follows SPIRIT guidelines [82] as outlined in a SPIRIT checklist.

## Methods: participants, interventions and outcomes

### Study setting {9}

The study is set in Kapilbastu district in Province 5 in the Western *Terai* (plains) of Nepal, bordering Uttar Pradesh state of India. The district population is 569,844 with an estimated crude birth rate of 21.3 per thousand population per year [83]. The population comprises predominantly *Madhesi* (plains) ethnicity Hindus with sizable minorities of disadvantaged Muslims and Dalits. Literacy rates are 45% and 65% amongst women and men respectively [83]. The lowland area is characterised by rice production with winter crops of wheat and pulses, with high temperatures and humidity for much of the year. Kapilbastu’s Human Development Index is 0.452, putting it in the second-least developed category of districts in Nepal [84]. Anaemia in women of reproductive age was 44% in 2016 [22].

#### Formative phase

In order to understand the project setting better and fit the intervention design to the context, we conducted formative activities before setting up the trial.

##### Policy engagement

We interacted with government stakeholders at federal, provincial and local levels, meeting with Ministry of Health & Population and Family Welfare Division, Province 5 Ministry of Social Development and Provincial Health Directorate, Kapilbastu Health Office, and elected municipality representatives. We orientated stakeholders about, and received their support for, trial activities around the time of the census, and whilst randomly allocating clusters to study arms. Before starting the interventions, we will orientate the municipal health team, health workers and FCHVs in selected clusters.

##### Formative research

We conducted a scoping review of literature to identify the current anaemia burden in PW, their dietary practices, health-seeking behaviour and research gaps for Nepal and South Asia. To explore the factors affecting compliance and consumption of IFA, access to antenatal care and consumption of micronutrient-rich food, we conducted a detailed qualitative study in two rural and one urban municipality of Kapilbastu.

We analysed pre-existing dietary data from LBWSAT in Nepal using ‘Optifoods’ linear programming software [85] to draw up dietary recommendations on the basis of available foods. The Optifoods analysis confirmed the difficulty in achieving an iron-replete diet using locally available foods, especially amongst vegetarians, but was helpful in identifying some key iron-rich foods to promote.

### Eligibility criteria {10}

#### Cluster selection

Prior to federal restructuring in 2017, the smallest geopolitical unit of administration in Nepal was the Village Development Committee (VDC), each divided into nine wards (hereafter ‘old wards’). Each old ward forms the catchment area of one female community health volunteer (FCHV) who is responsible for holding monthly health mothers’ group meetings in the community. Since these groups are the platform for our PLA intervention, we chose old wards as the basis for forming study clusters.

We estimated cluster population by applying World Bank annual population growth rates [86, 87] to Kapilbastu 2011 census data at the old-ward level [83]. On the basis of pregnancy detection rates in LBWSAT data, we predicted that 2.52 pregnancies/1000 total population could be detected per month per ward and conservatively assumed that up to two-thirds of the pregnancies detected would be > 20 weeks’ gestation, which would be too late to enrol into the trial.

Cluster inclusion criteria were set to ensure that the majority of participants will be from the population group with the highest anaemia prevalence in the district, which is Madhesi ethnicity rural women who make up the majority of the community in the south of Kapilbastu district. Hence, our cluster inclusion criteria are not adjoining the main East-West highway that traverses Nepal; lying in the southern part of Kapilbastu district (closer to the Indian border) where there is less population heterogeneity and lower forest coverage; in a rural area with no major market; projected population of ≥ 1100 and < 3200 from Nepal 2001 census; surrounded with a buffer zone of non-study clusters; and > 50% Madhesi (plains ethnicity) as per the pre-trial census (below) [88].

**Fig. 1:**
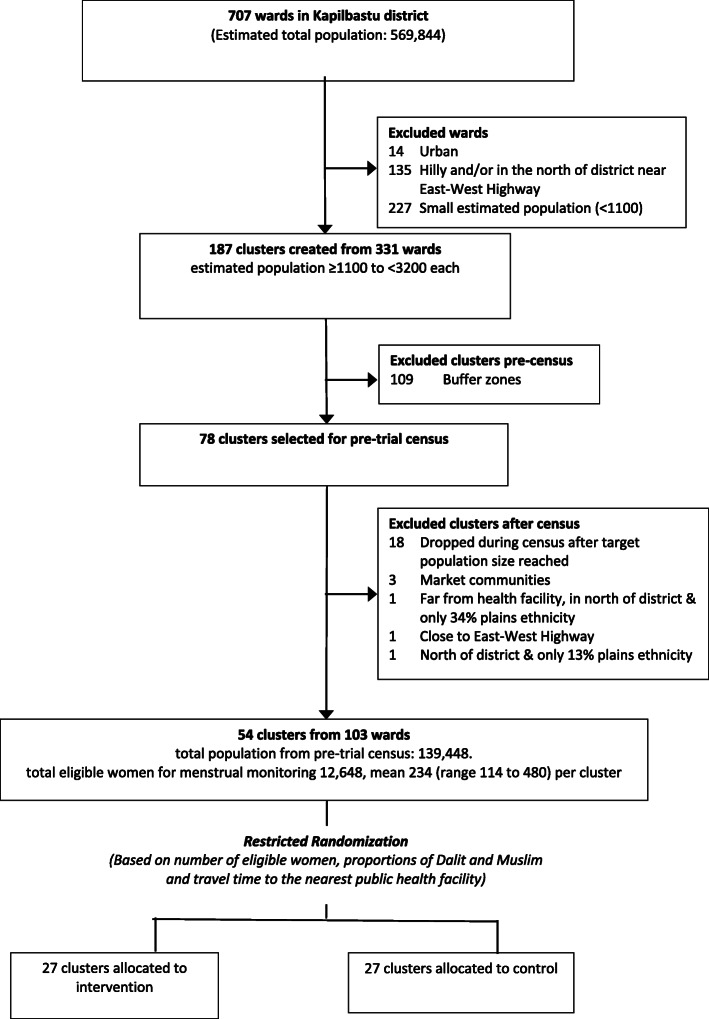
Flowchart of the process of study cluster selection

Figure 1 summarises the process of excluding old wards on the basis of population size and location within the district, merging them to come up with a sample of 78 clusters eligible for inclusion in the pre-trial population census, and exclusion of cluster post-census.

#### Pre-trial census

**Fig. 2:**
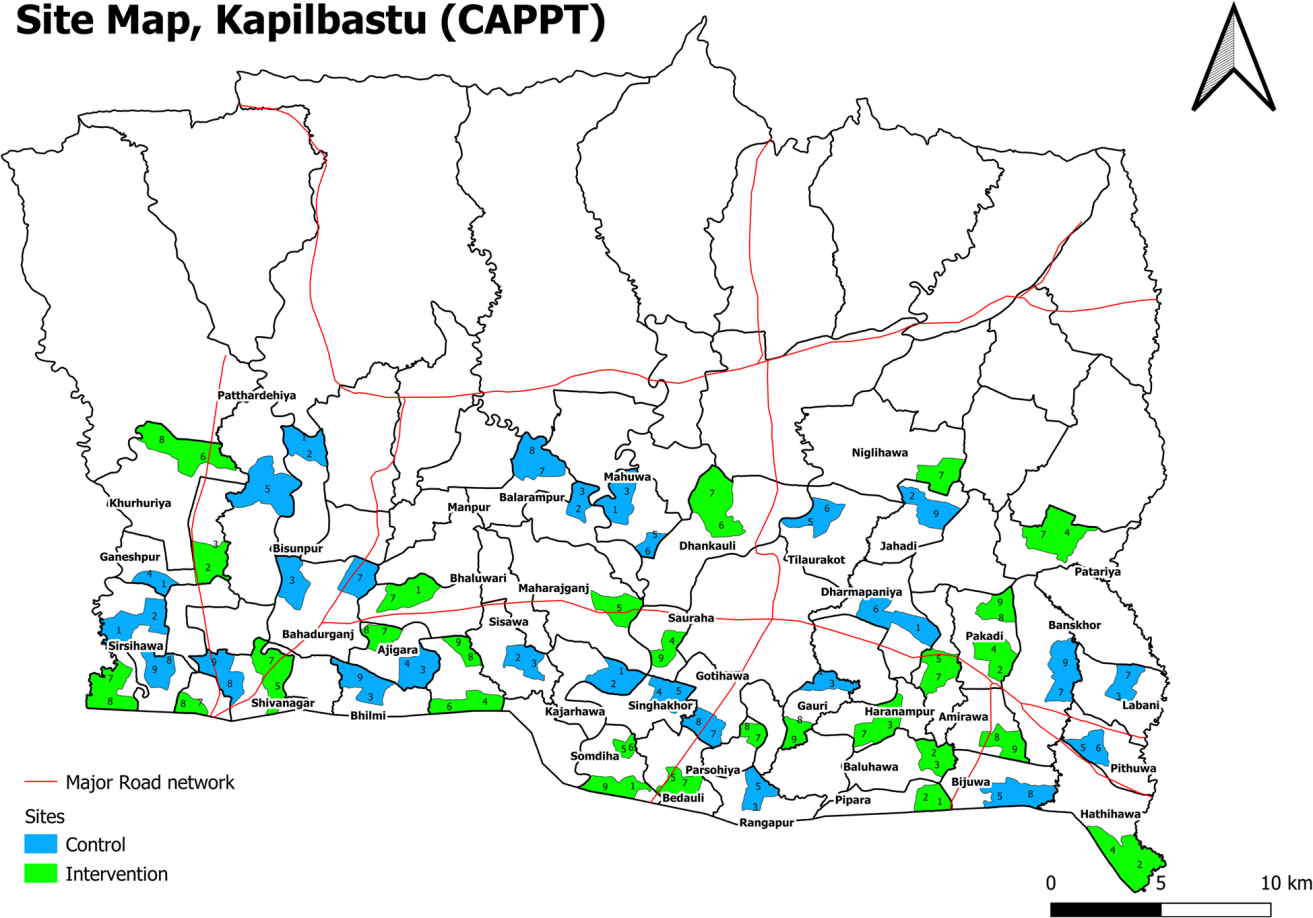
Map of study clusters showing randomly allocated clusters

From November 2019 to February 2020, we conducted a census to quantify the number of married women of reproductive age and describe cluster characteristics that could be used in restricted randomisation to allocate clusters to study arms such as caste, religion, household size, education and distance to the nearest public health facility. Although we originally planned to include all 78 eligible clusters, we decided to halt data collection after 60 clusters had been completed, since the population per cluster was considerably higher than predicted, dropping the 18 remaining clusters. The higher population per cluster also meant we needed only 54 clusters for the trial. Of the 60 clusters included in the census, we found that 6 were atypical on the basis of having a high proportion of hills ethnicity, being close to the east-west highway, far from the nearest public health facility or having a dense population with busy marketplace. Consequently, we dropped these clusters, resulting in the final selection of 54 clusters comprising 103 old wards (5 single- and 49 merged pairs of old wards), which have been randomly allocated to 27 clusters per arm, are listed in Supplementary Annex 2 and mapped in Fig. 2. The total population enumerated in the census is 139,448, mean 2582 (range 1299–4865) per cluster. The population of women identified as eligible for menstrual monitoring in the census is 12,648, mean 234 (range 114 to 480) eligible women per cluster.

#### Individual participant eligibility

Inclusion criteria to be enrolled into menstrual monitoring (to detect pregnancy) are being a married woman between the ages of 13 and 49 years: being resident in the study cluster (whether at her husband’s or parental home); could become pregnant (i.e. she and her husband have not had permanent sterilization such as tubal ligation or vasectomy, has not attained menopause nor had a hysterectomy); and consents to being asked about their menstrual status once every 4 weeks.

Inclusion criteria for a woman to be enrolled into trial follow-up include the following: a married female aged 13 to 49 years with a positive pregnancy test, at less than 20 weeks’ gestation, who is able to provide informed consent/assent and respond to survey questions. The gestational age is estimated from the date of the last menstrual period (LMP) as recalled by the PW, which is cross-checked against LMP dates recorded in the menstrual monitoring register during menstrual monitoring. If a PW has already visited a health facility for an ultrasound test, this report is also checked before enrolling the woman.

Exclusion criteria include non-consent and/or unable to respond to questions, ≥ 20 weeks’ gestation from LMP (or uterus clearly visible above the level of the umbilicus if LMP is not recalled/not available) and not planning to reside in the study cluster for most of her pregnancy.

Although the study participants give consent, their family members are also encouraged to participate in home visits and women’s groups. We will also ask for consent from husbands or adult male household members (or mother-in-law if no adult male), to measure their diets in addition to the PW.

### Who will take informed consent? {26a}

Letters of approval to work in selected clusters were received from municipality officials by field managers employed by HERD International before undertaking the census. Household heads provided written/thumbprint consent to collect census data.

All consent for individual participation (using signature or thumbprint) is taken by interviewers at the beginning of menstrual monitoring and again at enrolment into follow-up when pregnancy is detected. After obtaining written consent, interviewers take oral consent at subsequent menstrual monitoring and trial follow-up visits. A participant is free to withdraw consent or refuse data collection on any of these occasions. Interviewers will take written consent from the married woman herself where she is 18 years or above. For adolescents 13 to 17 years, interviewers will take written consent from guardians and written assent from the adolescent, and both shall be required for the girl to participate. Supplementary annexes 3, 4, 5 and 6 provide the trial participation information sheets and consent forms in English (copies in Nepali and Awadhi available on request).

### Additional consent provisions for collection and use of participant data and biological specimens {26b}

This trial does not involve collecting biological specimens for storage. Every consent form has a clause asking permission to share the anonymised data collected in this study with other researchers to conduct secondary analyses and to revisit the participant for future follow-up studies, should the need arise.

## Interventions

### Explanation for the choice of comparators {6b}

We compare intervention with control clusters, where women do not receive the interventions but have access to routine antenatal care services within the government health system. Menstrual monitoring in both intervention and control clusters may mean pregnancies are detected earlier, and women may receive ANC and/or IFA earlier, than outside the study area.

### Intervention description {11a}

#### Intervention staff recruitment and training

Six certified auxiliary nurse midwives are employed by HERD as nutrition assistants (NAs) to deliver tailored home visits to PW and facilitate PLA women’s groups in 4–5 intervention clusters each. Training of NAs will involve role-play practice sessions and field testing of intervention activities. Topics include (i) health consequences of anaemia in pregnancy and anaemia prevention/treatment; (ii) diet in pregnancy and how to increase iron intake and bioavailability; (iii) communication skills; (iv) how to engage families in dialogue and problem solving; (iv) use of Hemocue to measure Hb levels; (v) use of mobile phones/tablets and CommCare for recording and reporting of intervention activities; (vi) how to run women’s groups following a PLA intervention manual.

**Fig. 3:**
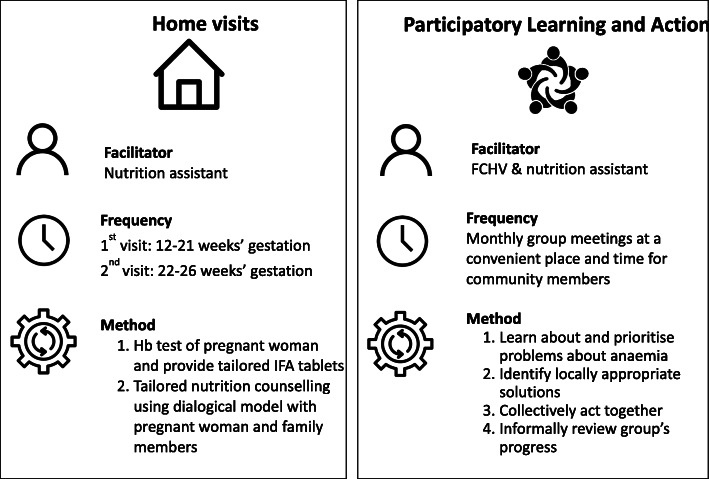
Components of the combined home visiting and Participatory Learning and Action (PLA) interventions

A diagram summarising the home visiting and PLA interventions is shown in Fig. 3.

#### Home visiting intervention

Home visits are designed to work synergistically to encourage PLA group attendance, to reach women who cannot / do not leave their homes and to engage family members. Each home visit will comprise dialogical counselling, home-based anaemia screening and tailored provision of IFA. The NA will visit each PW twice at home, first at 12 to 21 weeks and second at 22 to 26 weeks. Ideally the gap between visits will be 4 to 6 weeks, unless logistical constraints imposed by the COVID-19 pandemic disrupt activities.

##### Home-based tailored dialogical counselling

We will take a *dialogical* approach to engage pregnant women and their families to think critically about the causes of anaemia in pregnancy in their household and community. The NA will engage pregnant women and their families in a cycle of action and reflection: (1) listening for the key issues and emotional concerns of the household; (2) promoting participatory dialogue about these concerns and (3) planning and taking action about the concerns that are discussed. At the first and second home visits, the NA will use stories and inductive questioning to trigger dialogue and reflection [66]. Stories will directly address issues from our formative research. We will train the NA about common issues that may arise and provide a discussion and reference manual with examples of actions that pregnant women and their families could take. Families will make specific action plans to address the issues that are relevant for their family and in the second visit these will be reviewed, and a different story used to trigger discussion and reflection. The tailored counselling aims to support women and their families to take actions to change dietary practices, take IFA and deworming tablets, attend PLA groups and access antenatal care. If the NA observes any pregnancy danger signs, as per the government’s standard treatment protocol [45], she will advise the woman to seek care straight away from the nearest appropriate health facility.

##### Anaemia screening and tailored iron-folic acid therapy

At each home visit, following the nutrition counselling, the NA will measure the PW’s haemoglobin concentration using a hand-held Hemocue Hb 301+ analyser, measure her mid-upper arm circumference (MUAC) with a SECA tape (93/42/EEC) to assess thinness, and explain the results. Following GoN guidelines, the NA will advise the PW to take IFA as follows:

- Not anaemic (Hb ≥ =11 g/dL), one IFA tablet (60 mg elemental iron and 400 μg folic acid) per day.
- Mildly or moderately anaemic (Hb 7–10.9 g/dL), two IFA tablets (120 mg elemental iron and 800 μg folic acid) per day.
- Severely anaemic (Hb < 7 g/dL), the NA will immediately refer the PW for a blood transfusion at a higher health facility.

At visit 1 (at 12 to 21 weeks’ gestation), the NA will provide sufficient IFA tablets for a period of 4–10 weeks until her second visit, at 22 to 26 weeks’ gestation. For all women, NAs will emphasise the importance of increasing dietary diversity and consuming micronutrient-rich food. For women with low (< 230 mm) or very low (< 210 mm) MUAC, the NA will also emphasise the importance of consuming additional calories through more frequent and/or larger meals and taking adequate rest. For women with MUAC ≥ 300 mm, the NA will stress the importance of keeping active during pregnancy and avoiding sugary or fatty foods and large portions of rice.

After repeating the haemoglobin and MUAC measurements at the second visit, the NA will explain to the woman how her anaemia and thinness status have changed and provide the appropriate IFA dose. She will also assess compliance to IFA by asking the PW about their tablet consumption and checking used blister packs. At visit 2, the NA will provide a single (400 mg) albendazole tablet if the PW has not already received it as per Nepal’s national protocol for women in their second trimester.

The NA will record details of haemoglobin and MUAC readings, IFA and albendazole tablets provided on the Trial Participation Card (TPC) at each visit to enable participants to show health workers what treatment they have been receiving. The NAs will make two copies of discussion action sheets to record actions agreed to reduce anaemia based on the issues identified. One copy will be given to the PW’s family, and the other copy will be kept by the NA for reference. The NA will also record Hb, MUAC, IFA and albendazole given, and the actions agreed upon with the PW’s family on an electronic data collection form just after the visit is concluded. The NA will not enter data on the tablet or phone during the visit to allow fluid interpersonal interaction but will take a photograph of the TPC and the discussion action sheet before leaving the home.

**Fig. 4:**
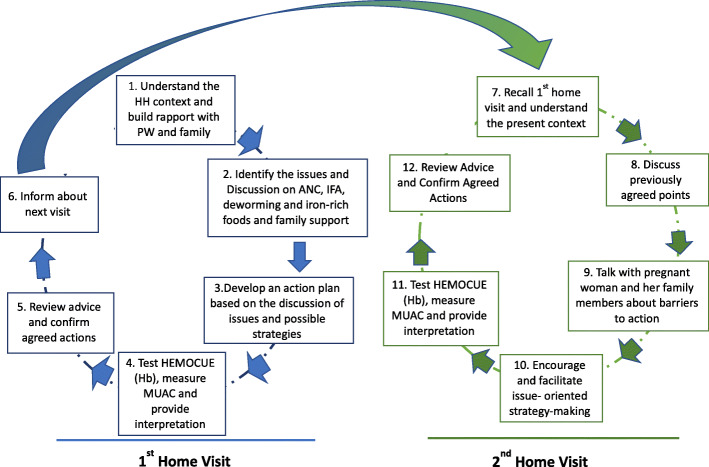
Modality of the home visiting intervention

The modality of the home visiting intervention is shown in Fig. 4.

#### Participatory Learning and Action women’s group (PLA) intervention

In the intervention arm, the NA, together with the local FCHV for that cluster, will facilitate monthly groups at a convenient place and time for community members. As FCHVs are mandated by the health system to hold monthly mothers’ groups, we will work through these groups where they already exist and revitalise them where they are inactive.

The groups will run for a 15-month period, from the last month of menstrual monitoring consent to (about 1 month before the first enrolment) until after the last enrolled woman has completed 30 ± 2 weeks gestation. NAs, who are locally recruited and trained, will train the FCHVs on the PLA meeting manual each month and the FCHVs will assist the NAs in group facilitation. Groups are open to anyone who is interested, and participation is voluntary. As restrictive gender norms can prevent women’s participation in mixed groups, the first 10 meetings will be exclusive to women. Men will be invited to a large community planning meeting and groups will discuss how they would like to engage with men thereafter.

The PLA cycle will consist of four phases (1) problem identification, (2) planning together, (3) strategy implementation and (4) strategy evaluation. During the ‘problem identification’ phase, over 6 monthly meetings, groups are introduced to the PLA method and discuss local definitions and beliefs about the causes and symptoms of anaemia, and local beliefs around taking IFA supplements. They will then discuss barriers to good nutrition to improve anaemia, and barriers to uptake of IFA supplementation during pregnancy. During the ‘planning together’ phase, over 3 monthly meeting groups prioritise problems that they would like to address and plan and implement a community meeting to engage the wider community. Groups will then lead on the implementation of these strategies in Phase 3 and will continue discussing new topics related to anaemia in pregnancy. They will evaluate the effect of their actions in Phase 4 by reflecting on the original problems and progress in solving them. Then they may reformulate strategies to begin another phase of implementation.

During the PLA cycle, facilitators use a pictorial meeting manual which contains varied triggers for discussion—such as a story, a quiz or game—which are often used with picture cards. These focus on iron- rich foods and how to increase bioavailability, improve IFA compliance, reduce side effects and manage nausea and minor pregnancy ailments, and when to seek care for more serious problems.

### Criteria for discontinuing or modifying allocated interventions {11b}

We do not expect that the tailored counselling or PLA women’s groups to have any negative effects upon trial participants, but some women may experience side effects from consuming IFA or from deworming. Side effects commonly experienced from IFA include constipation and indigestion [1, 89], but these tend not to be serious. Recent studies have also indicated that iron supplementation may increase susceptibility to infections but that this is more common in children than adults [90]. Interviewers will collect data on side effects of IFA and on morbidity of women during the 30 ± 2-week interviews and NAs will ask women about side effects and advise how to mitigate them during counselling sessions and at women’s groups. In exceptional cases, if the higher IFA dose is not being tolerated, the NA may advise the woman to reduce intake from two to one tablet per day. All women who are feeling unwell at the time of interaction with the NA or data collector are advised to seek care at their nearby health facility or at a higher care centre where needed.

### Strategies to improve adherence to interventions {11c}

Nepal’s national protocol is to provide PW with IFA from 20 weeks’ gestation onwards till 45 days post-partum from all health facilities, outreach clinics and FCHVs. Doubling the dose for anaemic women is in the GoN protocol but usually not practised. We will orient all health workers at the health facilities in/near intervention areas, and the district and regional hospitals, about the intervention and the distribution of IFA and albendazole tablets by the NA during home visits and encourage them to practice administration of double dose for women who have been identified as anaemic in line with NA prescription. NAs will ask women to show their home visit TPC records of Hb, MUAC, IFA and albendazole tablets to health workers at the beginning of each ANC consultation to ensure women are not prescribed double doses of IFA or albendazole. The NAs will visit health facilities every month and provide a list of the enrolled participants and the number of tablets provided to them to avoid duplication. For more distant health facilities, or where visiting health facilities is restricted by COVID-19, information is sent by email or SMS and NAs phone health workers to ensure the information has been received.

NAs attend monthly meetings with intervention coordinators to reflect on the previous PLA meeting and plan for the next. Through discussion and role-play, facilitators develop common methods of holding meetings. Whilst the trial surveillance system is getting established, a 1-month ‘run-in’ of women’s groups allows time for groups to get established before the first ‘full-trial’ PW enrols. The picture cards and women’s group manual can be modified as needed to ensure that content is realistic, understandable, culturally appropriate, visually appealing, and motivating, but any changes will be applied across all intervention clusters.

### Relevant concomitant care permitted or prohibited during the trial {11d}

Women who receive home visits and tailored doses of IFA and deworming are strongly discouraged from taking additional doses of micronutrients or additional deworming. NAs and interviewers encourage PW to seek concomitant care for any illnesses they may be experiencing which are reported during interactions. If the interviewer or NA detects severe anaemia, they give the PW a referral slip, and advise her to go immediately to the district hospital or other referral centre where transfusions are available.

### Provisions for post-trial care {30}

We do not envisage complications that would require compensation but have necessary insurance arrangements in place.

### Outcomes {12}

All outcomes are measured during 30 ± 2-week interviews and are listed in Table 1 below.

**Table 1:** Trial outcomes and indicators on the impact pathway

CAPPT trial outcomes	Effect measure to compare arms/summary statistic
**Primary outcome** at 30 ± 2 weeks	
Haemoglobin concentration ascertained from a Hemocue 301+ analyser reading (Hb g/dL)	Difference/mean
**Secondary outcomes** at 30 ± 2 weeks	
Prevalence of anaemia (% Hb< 11.0 g/dL)	Odds ratio/proportion
Mid-upper arm circumference (cm)	Difference/mean
Mean probability of adequacy (MPA) of 11 micronutrients including vitamin A, riboflavin (B_2_ ), niacin (B_3_ ), pyridoxine (B_6_ ), cobalamin (B_12_ ), thiamine (B_1_ ), folate (B_9_ ), vitamin C, iron, zinc and calcium;	Difference/mean
Total number of ANC visits a health facility	Ratio/mean
**Indicators on the pathway to impact to be compared between arms** at 30 ± 2 weeks	
IFA tablets consumed by time of measurement at 30 ± 2 weeks	Ratio/mean
Usual energy intake (kcal/day)	Difference/mean
Usual dietary iron intake (excluding supplements) (mg/day)	Difference/mean
Uptake of methods used to improve bioavailability	Ratio/mean
Nutrition knowledge score	Ratio/count

The primary outcome of the trial is mean haemoglobin concentration in g/dL ascertained from a Hemocue 301+ analyser reading. Secondary outcomes are prevalence of anaemia (% Hb< 11.0 g/dL), mid-upper arm circumference (cm) and mean probability of micronutrient adequacy of 11 micronutrients including vitamin A, riboflavin (B_2_), niacin (B_3_), pyridoxine (B_6_), cobalamin (B_12_), thiamine (B_1_), folate (B_9_), vitamin C, iron, zinc and calcium.

Indicators to compare between arms to assess pathways to impact include the following: count of ANC visits at a health facility, number of IFA tablets consumed during pregnancy, intakes of energy (kcal/day) and dietary iron excluding supplements (mg/day), a score of bioavailability-enhancing behaviours (comprised of avoiding tea and coffee at or near mealtimes, use of vitamin C-rich foods with food and IFA tablets, use of sprouted pulses or grains and spreading of haem-iron foods over 2 eating occasions), recall of nutrition knowledge indicators pertaining to iron-rich foods, importance of IFA and ways to improve bioavailability of iron.

To describe intervention implementation and potential mechanisms by which the intervention and its components may have an effect, we also collect process outcomes given in Table 2. Exposure to PLA groups, number of home visits and side effects will be reported in the main trial paper, together with factors which emerge as key indicators of intervention fidelity. Other process indicators may be reported in one or more separate publications. Process indicators include weight gain in pregnancy, gestational age at first ANC, amounts of promoted foods consumed, health literacy, social networks and social norms. For assessment of the effect of the intervention upon intra-household food allocation, we measure ratios of MPA, energy adequacy and iron intake between the PW and her husband (or senior household member).

**Table 2:** Process indicators

**Intervention exposure and activities**	
Number of home visits received (0, 1 or 2)^1^	
Family actions agreed upon in the home visit	
Which family members took part in the home visit interaction	
Doses of IFA prescribed	
Exposure to PLA community strategies^1^	
Whether any family members attended PLA groups	
Which family members attended	
**Social and behaviour change processes**	
Health literacy	
Social norms	
**IFA and ANC processes**	
Numbers of IFA tablets consumed in relation to those prescribed	
Uptake of any antenatal care	
Quality of antenatal care	
Gestational age at first ANC visit	
**Dietary processes**	
Amounts of key promoted foods consumed by pregnant women	
Intra-household allocation, as PW’s share {PW/(PW + senior male)} of nutrients in term of Mean probability of micronutrient adequacy, iron intake, and energy adequacy	
Weight gain in pregnancy from enrolment to 30 ± 2 weeks (kg)	
**Side effects**	
Constipation^1^	
Indigestion/heart burn^1^	
Vomiting^1^	

^1^These process indicators will be reported in the main trial paper whereas others may be reported in one or more separate process evaluation publications rather than in the trial paper

For the purposes of tracking any potential harms, we track side effects from taking IFA tablets including vomiting, constipation and indigestion or heart burn and report them to the DMC.

### Participant timeline {13}

**Fig. 5:**
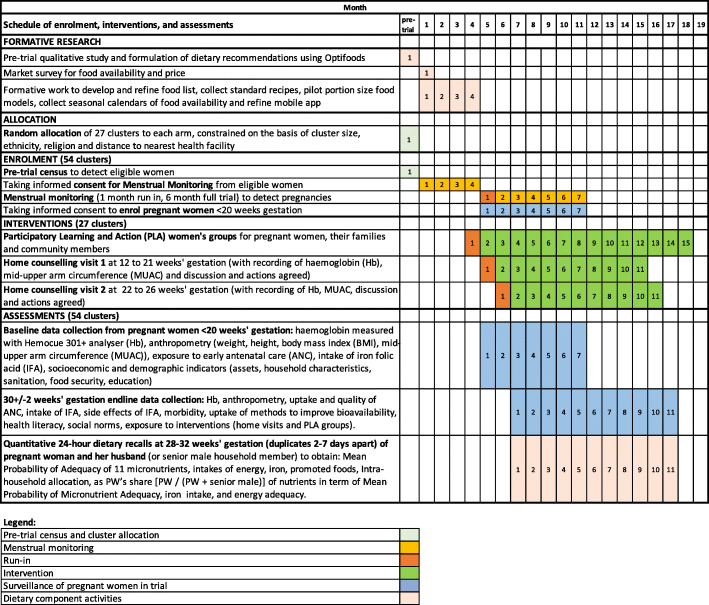
Schedule of enrolment, interventions, and assessments (SPIRIT Figure)

Formative work ran from May 2019 to February 2020 but trial roll-out was delayed because of the Novel SARS-CoV-2 Corona Virus (COVID-19) pandemic which began to affect Nepal in March 2020, just as trial enrolment was due to begin. Since census data is now out of date, several months of formal menstrual monitoring consent-taking and updating the census with new women or households is required before enrolment can begin. The trial timeline from when we are able to start the trial, showing the schedule of enrolment, interventions and assessments (as per the SPIRIT figure guidelines), is provided in Fig. 5. Data collectors will enrol women for one run-in and six full-trial months and follow-up every woman until the last enrolled woman reaches 30 ± 2 weeks. The ‘full-trial’ phase will last for 14 months and only women recruited after the run-in month will be included in the final analysis.

### Sample size {14}

Our trial includes 54 clusters with 6 months of enrolment. We assume 2.52 pregnancies will be detected per month per 1000 population, based on the rate observed in LBWSAT, which used a similar pregnancy detection mechanism. For women to be recruited, their pregnancy must be detected at < 20 weeks’ gestation. In LBWSAT, 55% of the pregnancies detected were < 20 weeks’ gestation at enrolment. So, for our sample size calculations, we assumed a best-case scenario of 50% and a worst case of 33% detected pregnancies < 20 weeks. We assume 20% loss to follow-up from enrolment to measurement of the primary outcome at 30 ± 2 weeks gestation, including losses due to miscarriage or termination and women migrating to their parental homes during pregnancy. Therefore, for the best- and worst-case enrolment scenarios, we expect an average of 15.6 and 10.4 primary outcome measures achieved per cluster respectively, giving us a sample size of 421 and 281 PW in each arm (or 842 and 562 women in the full trial).

We used Hb data provided by the Suaahara nutrition programme 2017 annual survey [91] from the neighbouring plains district of Rupandehi, to inform our sample size estimates. These data, which were sampled on the basis of old VDC wards that are the same as our cluster definition, provided a standard deviation (SD) of Hb of 1.2 and an intracluster correlation coefficient (ICC) of 0.09.

A Cochrane review showed a mean difference of Hb 0.888 g/L (95% CI 0.696 to 1.080) between PW supplemented with iron versus women without supplements [43]. Since in our control clusters we expect lower intakes of IFA and iron-rich food than in intervention clusters, we consider a 0.4 g/dL difference in Hb at 30 ± 2 weeks of gestation between trial arms plausible. We also consider this effect size to be of clinical importance.

Our power calculations are based on a difference of 0.4 g/dL Hb between arms in the primary outcome, an ICC of 0.09 and SD of Hb of 1.2 (or 1.25) g/dL and a coefficient of variation (CV) in cluster size of 0.27, based on CAPPT census data. We assume two-sided testing at the 5% significance level. In the best-case scenario of 50% of pregnancies being < 20 weeks’ gestation, the design provides 88% power assuming an SD of 1.2 and 86% power with an SD of 1.25. Full details of the power available in the different scenarios and details of the number of cases to be enrolled in the full trial (before loss to follow-up) are given in Table 3. Expected pregnancies and population per cluster used in power calculations are provided in Supplementary Annex 7.

**Table 3:** Power calculations based on different standard deviations and proportion of pregnancies detected < 20 weeks

% of pregnancies < 20 weeks^1^	No of clusters per arm	Average number of Hb outcomes per cluster	ICC (rho)	CV cluster size	Detectable difference in Hb	Power with SD of 1.2	Power with SD of 1.25
50%	27	15.6	0.09	0.27	0.4	88.3%	85.7%
33%	27	10.4	0.09	0.27	0.4	82.1%	79.0%

^1^enrolment cut-off is ≤ 19 weeks 6 days gestation

### Recruitment {15}

Strategies to achieve adequate participant enrolment to reach the target sample size include monitoring menstruation of all consenting non-pregnant eligible women in the cluster and a free urine pregnancy test (UPT) after one or more missed menstrual period so that pregnancies are enrolled before 20 weeks. We provide FCHVs a monthly travel allowance plus an additional incentive on the basis of pregnancies detected. We conservatively accounted for only 33 to 50% of pregnancies being detected < 20 weeks, 20% loss to follow-up, and will allow 1 month for trial run-in to iron out problems at the start. To increase the response rate in later pregnancy, we provide an incentive to trial participants (NPR 1000) at the 30 ± 2-week interview.

## Assignment of interventions: allocation

### Sequence generation {16a}

To randomly allocate clusters to two arms, we used covariate-based constrained randomisation, drawing upon census data to characterise clusters to enable a similar population composition between study arms. The trial statistician (AC) randomly generated 5000 potential allocations based on computer-generated random permutation, from which 4206 were rejected on the basis of any of the following thresholds for the difference in cluster mean between arms:

- Difference in % Muslim more than 5
- Difference in % hills ethnicity more than 2
- Difference in number of eligible women more than 17
- Difference in travel time to health centre on foot more than 3.5 min
- Difference in travel time to health centre by vehicle more than 2 min

These thresholds were set at approximately 0.25 of the SD in cluster summary value across the 54 clusters for each covariate and were considered adequate for good balance. We did not restrict the randomisation more strictly because of concerns this might impact on the type 1 error, for example as pairs of clusters tended to be allocated to the same arm. The remaining 794 candidate allocations were found unique and 12 were selected at random. A final list of 24 potential allocations was prepared, in which each of the 12 allocations was applied in the 2 possible ways (0/1 is either control/intervention or intervention/control) to ensure exactly equal chance of each cluster being allocated to control or intervention.

### Concealment mechanism {16b}

To ensure the randomisation process was observed to be fair, we held a meeting with municipality and health system leaders in March 2021 where we introduced the trial and invited a leader to randomly pick out the chosen randomisation option from a box of ping-pong balls labelled with the randomisation number. The allocation to arms was revealed to stakeholders and trial team members at the same moment in this public forum by a community stakeholder opening a sealed envelope with the chosen randomisation sequence and reading it out to the group.

### Implementation {16c}

The potential cluster allocation sequences were generated by the trial statistician (AC), and the final selection at the meeting (as described above) was made in advance of any recruitment of participants. Participants will be enrolled by field staff on the basis of meeting eligibility criteria and their cluster of residence determines allocation.

## Assignment of interventions: blinding

### Who will be blinded {17a}

After assignment of clusters to study arms, blinding of trial staff and participants is impossible, since the interventions are implemented at a cluster level and are publicised amongst community representatives, health workers and policymakers, to increase intervention uptake. Data collectors who collect primary outcome haemoglobin measurements and other secondary outcomes will know the study arm of the participant at the time of data collection, but since the Hb reading is a digital read out from a Hemocue analyser, the risk of bias in collection of the primary outcome is low.

### Procedure for unblinding if needed {17b}

The design is open label with only outcome assessors being blinded so unblinding will not occur. Interim reports for the Data Monitoring Committee (DMC) and Trial Steering Committee (TSC) regarding recruitment, follow-up and baseline characteristics are unblinded. Interim safety reports (morbidity and side effects) for the DMC will be initially blinded but can be unblinded on request of the DMC if differences are observed.

## Data collection and management

### Plans for assessment and collection of outcomes {18a}

**Fig. 6:**
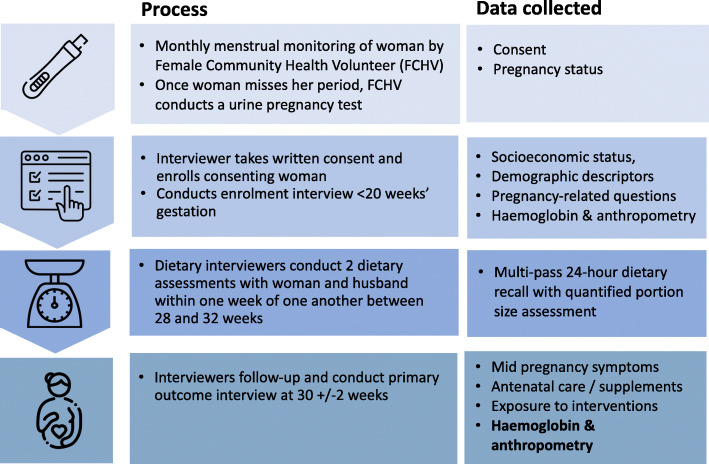
Surveillance system process

A chart outlining the surveillance system is provided in Fig. 6.

In total, 103 incentivised FCHVs, with support from 8 interviewers, will monitor menstrual status in paper registers every 4 weeks. Interviewers collect data on all trial participants at enrolment (< 20 weeks) and at 30 ± 2 weeks’ gestation, covering primary, secondary and process outcomes. Six specialised dietary data interviewers undertake two 24-h dietary recalls at 30 ± 2 weeks’ gestation on non-consecutive days but within approximately 1 week of one another. They interview the woman and her husband/or a senior adult male household member if the husband lives away. If there are no adult males, the mother-in-law (or other female household head) is interviewed.

#### Electronic data collection

Questionnaires are programmed in Nepali, Awadhi and English on android operating system tablets or mobile phones using the CommCare electronic data collection platform [92]. In-built jump-sequences and value limits prevent entry of data outside plausible ranges.

To enable seamless merging of data by participant and efficient case management and follow-up, unique identifiers (IDs) encoded into Quick Response (QR) codes are printed onto stickers. These are allocated to women enrolled by attaching them next to their names in menstrual monitoring registers. When a woman is enrolled in the trial, her QR code is attached to her a trial participant card (TPC) and to a trial participant list held by each interviewer. Her ID is scanned at every interaction with interviewers, dietary interviewers and NAs, enabling information stored in CommCare about that woman (such as name, LMP, and other details) to be ‘called up’ and used to ensure questionnaire flow and accuracy.

#### Standard operating procedures

To standardise haemoglobin concentration (Hb) measurements, anthropometric and 24-h dietary recall measurements, standard operating procedures (SOPs) are followed to ensure that measurements are accurate and inter-observer difference minimised. Hb is determined using a portable battery-operated electronic Haemoglobin Photometer (Hemocue Hb 301+, Angelhom, Sweden). The haemoglobin measurement follows standard procedures as per the manufacturer’s instructions. Anthropometric measures are taken following the WHO guidelines [93–95]. Mid-upper arm circumference (MUAC) and weight of women and their husbands are measured using Seca head circumference tapes (93/42/EEC) to the nearest millimetre, and Seca 877 weighing scales accurate to 100 g respectively. Heights are measured using portable Leicester Stadiometers. Calibration of Hemocue and scales are checked at least once per week by the interviewers. Videos on how to take anthropometric and Hemocue measurements have been developed for use during training and are installed on their tablets for reference. Interviewers’ tablets will be programmed to provide reminders for calibration checks and COVID-19 symptom checks as required. In the case of anthropometric measures, a third reading is required if the first two measures differ by more than a specified margin. Dietary intake assessments will follow standard protocols [96], including a five-stage multi-pass probing method to minimise underreporting [97].

#### Enrolment of trial participants

FCHVs are responsible for detecting pregnancies in their cluster as early as possible. Initially, the FCHVs and interviewers visit every identified woman to take her written consent for ongoing menstrual monitoring and to update information from the census (adding new women and households as appropriate). After consent has been taken in all clusters, trial enrolment starts. FCHVs record each consenting women’s LMP in the page of the register corresponding to her unique ID. The FCHVs subsequently visit every 4 weeks to record women’s menstrual/pregnancy status over a period of 7 months.

Once an FCHV finds a woman who has missed at least one period, she conducts a urine pregnancy test (UPT) at the woman’s house. If the UPT is positive, the interviewer visits the woman to check her eligibility and take her written consent to enrol in the trial. The interviewer provides each consenting eligible woman with a TPC with her unique QR code identification number affixed. If a woman is ineligible (≥ 20 weeks’ gestation, not planning to reside in the cluster, or unable to respond to questions), she is advised to seek ANC and, in the intervention arm only, is invited to join PLA women’s groups. In intervention clusters, interviewers notify the NAs about the location and gestational age of the newly enrolled eligible women to receive her first home visit and invite her to PLA groups.

When the interviewer takes informed written consent from women, they ask 5–6 questions to check the PW’s understanding of the trial process. Only those who can demonstrate that they have understood are enrolled. At both enrolment and primary outcome interactions, interviewers will measure the woman’s haemoglobin concentrations, height, weight and mid-upper arm circumference. We will also collect the woman’s age, parity, medical history, date of the last menstrual period, pregnancy symptoms/ problems, pregnancy intention as measured by the London Measure of Unwanted Pregnancy (LMUP), smoking and alcohol consumption, age of husband, household size and other socioeconomic and demographic information including caste/religion, education, landholdings and assets, and housing characteristics.

#### Outcome measurement in the third trimester of pregnancy

As listed in Table 1, all primary, secondary and process outcomes are measured at 30 ± 2 weeks. Although ideally during the 28-to-32-week window, due to potential disruption that the ongoing COVID-19 pandemic may cause, an outcome may be collected any time up to delivery as gestational age at measurement will be adjusted for in all analyses.

Dietary interviewers use an adapted quantitative 24-h dietary recall tool [98] to measure dietary intakes of PW and their husbands or another ‘senior’ male (or a female household head if there are no men). Recalls are taken twice on non-consecutive days after between 28 and 32 weeks, including typical and atypical (celebratory and fasting) days. Daily nutritional intakes are calculated using (i) a list of locally available foods; (ii) quantified intakes of each food, estimated using a combination of weighed methods (using food models) and a photographic atlas of graduated portion sizes [99] and (iii) a Food Composition Table that integrates nutritional composition data from Nepal [100–102], India [103], Bangladesh [104], USA [105], UK [106, 107] and published back-of-pack information from local foods [102].

#### Monitoring and supervision of data collection

All interviewers are supervised by field coordinators and a monitoring manager who observe 5% of interviews, take replicate measures of anthropometry and monitor electronic form submissions. The monitoring team meets monthly and Kathmandu-based team members visit and/or hold video-conferencing meetings periodically to provide support. Problems with electronic forms are logged and corrections made reversibly in the data using Stata data cleaning ‘do’ files. A Kathmandu-based data management team monitors data daily.

### Plans to promote participant retention and complete follow-up {18b}

Strategies to promote participant retention include collecting and updating phone numbers of respondents so interviewers can arrange a convenient time to visit; and providing a small gift at the end of data collection. Within the CommCare forms, target dates for intervention and follow-up events, based on the LMP, are displayed on customised lists by user to help interviewers follow the schedule.

### Data management {19}

Data are stored in the following places:

- FCHVs hold paper registers for menstrual monitoring
- Interviewers have lists of women enrolled in menstrual monitoring permanently stored on password-protected tablets, and survey data are temporarily stored before they are synchronised (encrypted) to a CommCare cloud server. Essential information required for case management is retained on the devices within the CommCare to facilitate follow-up and questionnaire flow.
- Enrolled women have a copy of some of their own data, recorded on their trial participant cards
- Health facilities are provided with enrolled women’s names and the IFA and albendazole prescribed by NAs.
- HERD International’s data management team in Kathmandu downloads all new data daily from the CommCare cloud server onto their server using a semi-automated system to extract csv files, import them into Stata and run automated do files for data pseudonymising, labelling and recoding.

Pseudo-anonymised data are shared with other data team members as needed but the person-identifiable information is stored in separate encrypted files, which are not used day-to-day unless follow-up lists need to be generated or maintained by authorised team members. Follow-up lists and data collector performance outputs are uploaded to a shared folder for the field managers and data coordinators to perform their checks.

All data are stored on password-protected, encrypted, secure server computers in lockable rooms at the Kathmandu office. Data on physical servers are backed up daily onto secure cloud servers and copied onto external secure backup hardware-encrypted hard drives in Kathmandu each week. The pseudonymised files are shared with named analysts / data managers for inspecting data quality and generating data summaries for the Data Management Committee and Trial Steering Committee as appropriate. Study arm is encoded but not labelled. Once all of the data have been collected and uploaded to the secure server and follow-up is complete, the data are deleted from supervisors’ laptops and data collection tablets, and eventually from the CommCare server.

Qualitative data collected in Awadhi and Nepali languages are transcribed in Nepali. After transcription, qualitative data collectors send audio recording and transcriptions to the HERD Kathmandu office where core team members check completeness and anonymisation and store the data in a lockable cabinet. The transcribed anonymised data is then translated to English, cross-checked with the original Nepali transcripts and thematically analysed.

#### Data cleaning

Data cleaning is completed by HERD International Data Management teams, NS and HHF as required. Variable naming, labelling and initial recoding is automated at the time of downloading. For all outcomes and important process variables and covariates, variable distributions are checked for normality and skewness and outliers identified and removed as necessary.

#### Data archiving

After the trial is complete, the pseudonymised trial master file, including all datasets, is securely stored electronically with trial partners in HERD, UCL and LSHTM. Both pseudonymised and person-identifiable data are uploaded to the UCL Data Safe Haven along with the questionnaires, data codebook and brief description of the trial. Archiving of data in Nepal follows HERD International’s and NHRC policies.

The pseudonymised data will be made open access using the UCL data sharing platform or similar as per MRC guidelines. Any request for archived person-identifiable data will go through the trial management team.

### Confidentiality {27}

It is necessary to obtain and maintain lists of participants’ names to enable women to be followed-up to detect pregnancy and track their progress through pregnancy. It is also necessary to communicate to local and health facilities women’s receipt of IFA and deworming at home visits, to prevent double dosing. However, person-identifiable information (names of household members and GPS location) will only be retained by the field staff who need to seek to interact with trial participants and this information will not be made available to others. Apart from follow-up lists, which interviewers and home visitors will utilise, all other data stored on portable, or team members’ computers are anonymised such that the participants are identified by their unique ID number but not their name, address or geolocation.

Person-identifiable information associated with trial participants such as their name and geolocation and signed consent forms are stored in separate encrypted data files in a UCL data safe haven and on encrypted and password-protected external media which are stored in locked cupboards kept by the trial PIs and data manager in HERD.

During the consent process, study participants are assured that all person-identifiable information will not be attached to the data used for analysis. They are also assured that the information they share with FCHVs regarding their menstrual or pregnancy status and any details recorded on data forms will not be shared with anyone in the community. Participants consent to their names and contact details being kept on follow-up lists on phones, tablets, computers, registers and paper lists but that this information are accessible only to specified members of the data management team and to the field team members that need to visit them during follow-up. They are told that the details of any IFA and deworming provided to them are shared with local health facilities to avoid double dosing. They understand that they will not be identifiable in the data that is used for the study analysis or in any data shared within and beyond the study team.

If a participant withdraws from the study after data are anonymised and shared for analysis, it may not be possible to remove their data, but we can assure them that they can be removed from follow-up lists in the future, if they do not wish to be contacted.

### Plans for collection, laboratory evaluation and storage of biological specimens for genetic or molecular analysis in this trial/future use {33}

No storable biological specimens are collected during this study.

## Statistical methods

### Statistical methods for primary and secondary outcomes {20a}

A detailed Statistical Analysis Plan, drawn up by the trial statistician (AC) and Nepal principal investigator (NS), will detail the analysis strategies, covariate adjustment and the approach to any missing data. Each version is presented to the Data Monitoring Committee (DMC) and Trial Steering Committee (TSC) for approval and the first version is prepared before the first patient is recruited.

Primary analysis is by intention-to-treat and will use the standard 5% significance level. Analysis of the primary outcome (haemoglobin concentration at the 30 ± 2 weeks) is based on linear regression, with random effects to adjust for clustering. Analyses are conducted with and without adjustment for predictors of the primary outcome (Table 4) including socioeconomic status, parity, age of PW, gestational age at measurement, maternal education and characteristics used in the restricted randomisation (individual level religion, ethnicity and travel time to the nearest health facility and cluster-level number of eligible women from the pre-trial census).

**Table 4:** Variables to adjust for or to use in subgroup analyses and exposure variables

**Confounders/covariates to adjust for in analyses:**	
Wealth score constructed out of household assets	
Education status of pregnant woman (none, primary, secondary, higher secondary +)	
Parity (*n*)	
Age of pregnant woman in years	
Gestational age at measurement in weeks	
Baseline (study enrolment) measure of Hb (for primary outcome analysis)	
**Adjustments for study design**	
Religion (Muslim versus Hindu)	
Ethnicity (hills versus plains)	
Travel time to health facility	
Cluster size (number of eligible women identified in the cluster during the census as used during constrained randomisation)	
Cluster (as random effect)	
**Variables to use in subgroup analyses**	
Baseline (study enrolment) anaemia status	
Categories of BMI at enrolment (kg/m^2^)	
Wealth categories constructed out of household assets	
**Main exposure (independent) variable for analyses of outcomes above:**	
Study arm: Home visiting +PLA versus Control (which is the reference group)	
**Within the intervention arm the levels of exposure to PLA women’s groups and home visits are coded in three (independent) variables for analyses of a dose-response effect upon the outcomes above (or a subset of them):**	
Exposure to women’s group—score of exposure constructed out of number of meetings attended by pregnant woman and number attended by family members	
Exposure to home visiting with tailored counselling and dose of iron-folic acid supplements—score of exposure constructed out of number of meetings attended by pregnant woman and number attended by family members	
IFA consumption during pregnancy (count or ordinal score constructed from the count)	

Our primary analysis will also adjust for haemoglobin at enrolment, to gain precision, but we acknowledge the trade-off that this may dilute intervention effects since in principle cluster-level intervention effects could be mediated through haemoglobin at enrolment. In a sensitivity analysis, we will remove adjustment for haemoglobin at enrolment.

Analysis of secondary and impact pathway outcomes (Table 1) and of process indicators (reported in 1 or more separate publications (Table 2)) will follow a similar methodology. We will use linear regression for continuous outcomes, logistic regression for binary outcomes, ordinal logistic regression for ordinal outcomes and negative binomial regression for ‘count’ outcomes such as number of IFA consumed. For continuous and count outcomes, before analysis of intervention effectiveness, the distribution will be examined and in the event of skewed or heaped data transformations such as log will be considered, or methods will be adapted such as for zero-inflation. For energy, iron and other micronutrients, which are (almost) ubiquitously consumed, we will use our duplicate measure to account for the wide intra-individual variability with a nonlinear random effects model and person-specific random effects (the National Cancer Institute method). For intakes of recommended foods that are only episodically consumed but also have wide within-person variance, we use a two-part model where the probability of consumption is estimated using a multilevel logistic regression, the amount consumed on consumption days is estimated by fitting a multilevel nonlinear regression model and the error terms of the two parts are correlated [108].

#### Process evaluation

**Fig. 7:**
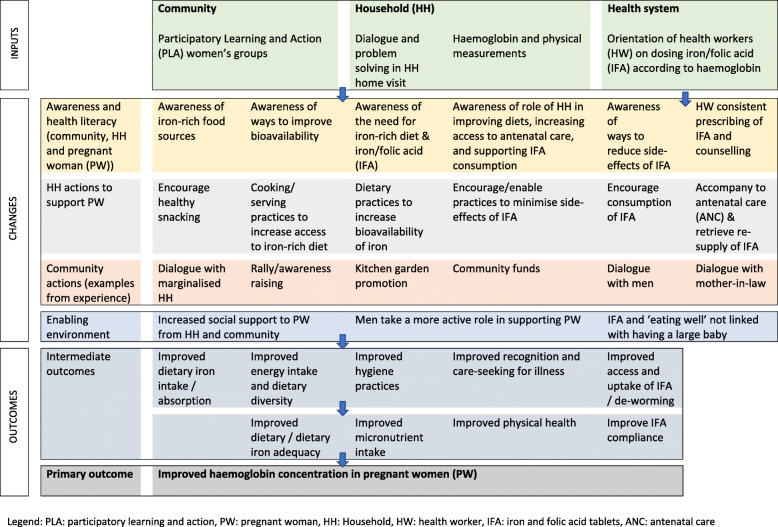
Theory of change

Our process evaluation is informed by MRC guidance and our theory of change (Fig. 7), and captures how the intervention was implemented, fidelity to plans, reach of the intervention, pathways to impact and contextual factors that explain heterogeneity in effects. The Theory of Change was developed through two workshops and refined after reviewing formative research findings. It details the hypothesised mechanisms which will enable change in our primary and secondary outcomes in intervention areas and the assumptions that underpin them.

Quantitative process outcomes, captured through surveillance questionnaires, have been included in Table 2. We explore how the context in both intervention and control areas may influence intervention effect [109]. We will describe the implementation of the intervention and fidelity to plans through analysis of process monitoring forms administered by supervisors of NAs. A senior process evaluation officer will review these data, and conduct focus group discussions with supervisors and NAs three times during through the intervention to explore how context affected the implementation of the intervention, and to analyse factors affecting attendance at groups and exposure to home visits. If we find effects on our primary outcome, we will use mediation analyses to unpack pathways along our theory of change, exploring whether effects are explained by changes in health literacy, social norms, dietary iron intakes, other micronutrient intakes, IFA compliance or deworming.

#### Economic evaluation

Cost and cost-effectiveness of the intervention will be estimated from a provider perspective. The costs of design and implementing the intervention package (programme costs) and costs to public health care providers will be estimated. Programme cost data will be collected from the project accounts of the implementing partner, staff time use surveys and interviews with project staff. Costs to public health care providers as a result of any increased demand for health services caused by the intervention, will be estimated using health seeking data from the 30 ± 2-week questionnaires with the study participants and available secondary data on unit costs for the services in question. Incremental cost-effectiveness of the intervention package will be calculated as compared to routine care. Incremental cost-effectiveness ratios (ICERs) will be estimated for the primary outcome and for associated disability-adjusted life years (DALYs) averted. A series of sensitivity analyses will be conducted to check the robustness of the results.

### Interim analyses {21b}

No interim analyses of intervention effectiveness are planned. Interim reports for the Data Monitoring Committee (DMC) and Trial Steering Committee (TSC) regarding recruitment, follow-up and baseline characteristics will be prepared and interim safety reports (morbidity and side effects) for the DMC.

We do not specify a formal stopping rule since the interventions under test are simply adapting WHO and government policies for IFA supplementation that are already in use. So, whilst women may consume more IFA tablets as a result of the intervention, there is no new drug being tested. The risk during the finger prick test for Hemocue testing to know the haemoglobin status is low. We also do not intend to consider modification or early stopping of the trial on the basis of interim evidence of intervention effectiveness (or lack of effectiveness). Nevertheless, the DMC is presented with harms data (adverse events and levels of morbidity) disaggregated by study arm at each meeting and could recommend to the Trial Steering Committee modification or termination of the trial.

### Methods for additional analyses (e.g. subgroup analyses) {20b}

Subgroup analyses will compare the intervention effect on the primary outcome by socioeconomic status, BMI category at enrolment and by baseline anaemia status (Table 4). The intervention effect is presented within subgroups and testing for differential effect by subgroup is based on an interaction term, using the same regression approach as for our main analyses.

Our main analyses are under the intention-to-treat principle, i.e. as randomised at the cluster level regardless of uptake of home visits, tailored IFA dosage or PLA. However, for the primary outcome, we will also conduct a ‘per protocol’ analysis in which participants who received the intervention only (i.e. who had PLA groups running in their community and received at least one home visit) are compared to all participants in the control arm.

Within the intervention arm, we will analyse the dose-response effect upon the primary outcome (Table 4). Three separate exposure scores will be derived based on the (1) number of home visits received, and number of family members engaged during visits, (2) number of PLA meetings attended by PW and number attended by family members and (3) number of IFA consumed. We will investigate the association between each score and the primary outcome, with and without adjustment for the other scores.

### Methods in analysis to handle protocol non-adherence and any statistical methods to handle missing data {20c}

We expect very low missing data in our baseline covariates and in the haemoglobin measure at enrolment. We do anticipate some women will not have a primary outcome value. Where this is due to miscarriage, we do not regard this as ‘missing’, but where the woman is unavailable or has moved away, we consider this to be missing. We do not however consider that imputation of the primary outcome is helpful since there is little information on which to base the imputation other than baseline/enrolment measures, which we are including as covariates in our regression models.

### Plans to give access to the full protocol, participant-level data and statistical code {31c}

Two versions of the trial protocol are publicly available, a version published on the ISRCTN registration page, and this published protocol.

Participant-level data will be shared only at the time of the publication of the main trial paper but will be made fully available subsequently. Statistical code for conducting the trial analysis will be shared in a web annex to the main trial paper.

## Oversight and monitoring

### Composition of the coordinating centre and trial steering committee {5d}

The coordinating centre for the trial is the Trial Management Group comprised of senior representatives from HERD International, the PI / local PI from UCL, the trial statistician and key personnel involved in the day-to-day management of activities on the ground. The TMG meet weekly during trial set-up period and two-weekly at other times.

The Trial Steering Committee is comprised of experts from various institutions in Nepal, the UK, India and Canada who are specialists on maternal nutrition, anaemia, haematology, conduct of trials and/or maternal nutrition in Nepal. The role of the TSC is to provide oversight for the trial, providing advice through its independent Chair to the Trial Management Group (TMG), MRC and/or any other Funder on all aspects of the trial. The TSC will meet at least yearly, but interim meetings will be organised as required, in person or by teleconference (or by a combination of the two). Certain trial issues may need to be dealt with between meetings, by phone or by email. Full details of the TSC charter and membership are provided in Supplementary Annex 1.

### Composition of the data monitoring committee, its role and reporting structure {21a}

The data monitoring committee is made up of experts from the fields of global health trials, statistics, obstetric haematology, maternal nutrition, micronutrient status and research ethics in Nepal, with three members from Nepal and the remaining three from the USA, UK and India. The role of the DMC is (i) to protect and serve the CAPPT trial participants and to assist and advise the Principal Investigators so as to protect the validity and credibility of the trial; (ii) to safeguard the interests of the trial participants, assess the safety and efficacy of the interventions during the trial and monitor the overall conduct of the trial. The DMC is entirely independent of the sponsor and all members declare that they have no competing interests.

Meetings involve members and investigators who are outside Nepal joining by video link so as to reduce costs and carbon emissions. The DMC meets once to review and approve the protocol and once towards the end of the trial to review trial findings. Further interim meetings may be called if needed and the investigators may seek advice from the chair by email if the situation requires. After each meeting, the DMC chair will prepare a brief report of DMC recommendations which is signed and dated and shared with the TSC chair, the Trial Statistician and the co-PIs within 3 weeks of the meeting. The charter of the Data Monitoring Committee is provided in Supplementary Annex 8.

### Adverse event reporting and harms {22}

Surveillance questionnaires and questions posed during counselling sessions will assess side effects of taking IFA which we expect will mostly be mild symptoms including dark stool, nausea, bloating, abdominal discomfort, heartburn, loss of appetite, metallic taste and constipation. The most serious problems that could arise may be a severe allergic reaction to IFA (though this is very rare) or participants contracting COVID-19 after interacting with a trial team member. In addition, our primary outcome questionnaire will record recalled symptoms of illness including pre-eclampsia, vaginal bleeding, dysentery, gestational diabetes and malaria, though none of these are expected to be associated with our intervention.

Although we do not expect any adverse effects of attending a PLA group, of being visited for counselling or interviews/measurements, we will establish a complaints procedure and ensure that trial participants and their families know who to call or where to visit to register a complaint. Contact details are provided in the participation information sheet, and participants will be reminded of the complaints procedure at each visit.

If any unanticipated effects (including maternal deaths or COVID-19 cases associated with trial participation) are noted by the NAs, interviewers or dietary data collectors, they will inform field coordinators / managers who will telephone and/or visit the home of the respondent to ascertain the extent and nature of the problem and complete an adverse events form. Investigators will then classify each event as a minor adverse reaction (MAR), severe adverse reaction (SAR), severe adverse event (SAE) or suspected unexpected serious adverse reaction (SUSAR). These will be presented to PIs and to the Data Monitoring Committee where appropriate. In the case of illness, all participants are referred to the appropriate referral centre depending on the severity of their condition.

#### Harms associated with COVID-19 and their mitigation

The COVID-19 pandemic raises new issues with respect to potential harms to study participants and /or to research team members. Depending on the levels of COVID-19 in the community, visiting women in their homes and calling community groups together may incur risk of increasing the spread of the disease, putting participants, trial implementers, their families and communities at risk. The measurements that need to be taken in interviews and during home visits require physical contact. PLA groups require communities to gather together, often in quite cramped conditions to keep out of the sun or rain, which makes keeping a distance of 2 m between group participants impossible.

Because of these risks, the CAPPT trial enrolment and follow-up was delayed between March 2020 and February 2021. Preparations were cautiously being initiated for enrolment to start in June 2021, but these were halted due to a new devastating wave of COVID-19 which began to affect Nepal in April 2021.

We have devised adapted SOPs for COVID-19 Infection Prevention and Control (IPC) involving wearing of a masks by both field team members and study participants at all times during interactions, washing hands with soap and water (or sanitiser) on arrival at a local and before departing, sanitising all anthropometric equipment (stadiometers, weighing scales and MUAC tapes) and Hemocue with antiseptic solution between every use and maintaining 2 m distance wherever possible, except when taking readings. For use of picture cards and photographic manuals for data collection, participants will be encouraged to use a stick to indicate pictures rather than touching them. If picture cards are passed around at all, they will be laminated and wiped clean with alcohol between interactions.

Before going to the field each morning, each staff member fills a COVID-19 symptom data collection form. They also phone the respondents that they are planning to meet that day to fill in the symptom form with them as well. If any symptoms of fever, new cough, anosmia (loss of sense of smell) or any new difficulty breathing are observed or have been observed within the last 14 days in the trial staff member or study participant, or any of their households then the person is considered a suspected COVID-19 case. We also check if anyone has tested positive in the last 14 days. In the case that any of the above are positive, the interaction will be postponed until the 14-day threshold is reached. After each 14-day period of non-contact, the COVID-19 screening form is repeated until it is safe for the interaction to be undertaken. If infections increase, this may affect the timing of enrolment and follow-up visiting and the fidelity of the intervention to plans.

### Frequency and plans for auditing trial conduct {23}

The trial management committee regularly reviews progress of the trial against the timeline and target sample size by arm. Additionally, the trial steering committee and data monitoring committee conduct independent progress reviews (to which the funder (MRC) is invited), as per their Terms of Reference.

### Plans for communicating important protocol amendments to relevant parties (e.g. trial participants, ethical committees) {25}

Any deviation from the protocol is documented and reported to the Principal Investigator, Sponsor and all other research partners immediately. If major changes to study design are needed during the trial, we will send an amended protocol to the Trial Steering Committee for approval and will seek approval from the NHRC, UCL and LSHTM ethics boards. We will also amend the entry in the trial registration registry and publish an amendment to this published protocol as follows:

**Table:** 

**Amendment No.**	**Protocol version no.**	**Date issued**	**Author(s) of changes**	**Details of changes made**

### Dissemination plans {31a}

In addition to publications in peer-reviewed journals, national- and provincial-level dissemination workshops will be held in Nepal with relevant policy makers, government officials, academics and other relevant stakeholders to inform them about the trial results. A policy brief will be prepared in English and Nepali and distributed widely at the dissemination events and elsewhere. Trial investigators will seek funds to present findings of the trial and of any ancillary studies in national, regional and international conferences or workshops wherever possible.

## Discussion

We hope that the combination of PLA community groups with two home visits to pregnant women with dialogical counselling and tailored dosing of IFA will result in improved haemoglobin levels, better dietary intakes and better nutritional status amongst pregnant women. Whether our intervention package is effective or not, the evidence generated by this trial will inform policy and practice to reduce anaemia in pregnancy in Nepal and elsewhere in South Asia.

## Trial status

Protocol version number Version 1.4. Date: 15 Jan 2022

Recruitment commencement date: recruitment was planned to start in June 2021 but the COVID-19 pandemic in Nepal has made implementation as planned impossible for the time being. Hence, we plan to begin recruitment on 1 July 2023, pandemic scenario and funds permitting.

Recruitment completion date: If the pandemic permits recruitment to begin on 1 July 2023. We will complete enrolment approximately 6 months later (by 28 Feb 2024).

## Supplementary Information

**Additional file 1: Supplementary Annex 1.** Trial Steering Monitoring (TSC) charter.

**Additional file 2: Supplementary Annex 2.** List of selected study clusters with population and expected pregnancies.

**Additional file 3: Supplementary Annex 3.** Menstrual monitoring participant information sheet in English.

**Additional file 4: Supplementary Annex 4.** Full Trial participant information sheets in English.

**Additional file 5: Supplementary Annex 5.** Menstrual monitoring consent form in English.

**Additional file 6: Supplementary Annex 6.** Full Trial consent form in English.

**Additional file 7: Supplementary Annex 7.** Expected pregnancies to be enrolled and numbers after loss to follow-up.

**Additional file 8: Supplementary Annex 8.** Data Monitoring Committee (DMC) charter.

## Abbreviations

ANC: Antenatal care
CAPPT: Comprehensive Anaemia Programme and Personalised Therapies
DHS: Demographic and Health Survey
FCHV: Female Community Health Volunteer
GoN: Government of Nepal
Hb: Haemoglobin
HERD: Health Research and Social Development Forum, Nepal
ISRCTN: International Standard Randomised Controlled Trial Number
IDA: Iron deficiency anaemia
IFA: Iron-folic acid supplement
LMP: Last menstrual period
LMUP: London Measure of Unwanted Pregnancy
LSHTM: London School of Hygiene and Tropical Medicine
LBWSAT: Low Birth weight South Asia Trial
LMICs: Low- and middle-income countries
MPA: Mean Probability of Adequacy
MRC: Medical Research Council
MUAC: Mid-upper arm circumference
MoHP: Ministry of Health & Population
MICS: Multiple Indicator Cluster Survey
NHRC: Nepal Heath Research Council
NNMSS: Nepal National Micronutrient Status Survey
COVID-19: Novel SARS-CoV-2 Corona Virus
NA: Nutrition Assistant
NEC: Nutrition Education and Counselling
PLA: Participatory Learning and Action
PW: Pregnant woman / women
QR code: Quick Response code
RCT: Randomised controlled trial
SOP: Standard operating procedure
TPC: Trial participant card
TSC: Trial Steering Committee
UCL: University College London
UPT: Urine pregnancy test
VDC: Village Development Committee
