# Supplementary material for: Comprehensive Anaemia Programme and Personalized Therapies (CAPPT): protocol for a cluster-randomised controlled trial testing the effect women’s groups, home counselling and iron supplementation on haemoglobin in pregnancy in southern Nepal
Source: Trials. 2022 Mar 1;23:183. doi: 10.1186/s13063-022-06043-z (PMC8886560; doi:10.1186/s13063-022-06043-z)
Supplement: Supplementary file 1 — Additional file 1: Supplementary Annex 1. Trial Steering Monitoring (TSC) charter. [file 13063_2022_6043_MOESM1_ESM.docx]

**Supplementary Annex 1: Trial Steering Monitoring (TSC) charter**

**CAPPT**

**Trial Steering Committee Charter**

[Comprehensive Anaemia Program and Personalised Therapies Trial]

Two sister non blinded cluster randomized controlled trials conducted in rural areas in Nepal and India

ISRCTN registration of Nepal trial: ISRCTN 12272130

Trial Steering Committee Charter

Version 2, Date 24.02.2020

(developed using MRC Clinical Trials Unit template TSC Charter version 1.02, 13-Mar-2006)

| **Authorised by:** | |  |  |  |  |
| --- | --- | --- | --- | --- | --- |
| Name: | [Sara Hillman] | |  | Role: | [Chief Investigator] |
| Signature: |  | |  | Date: | 23-12-2019 |
|  | | |  |  |  |
| **Prepared by** | | |  |  |  |
| Name: | [Sara Hillman] | |  | Role: | [Chief Investigator] |
| Signature: |  | |  | Date: | 23-12-2019 |

| Content | | details of TSC |
| --- | --- | --- |
| Name (& Sponsor’s ID) of trial | | All India Institute Medical Sciences, New Delhi and HERD, Nepal  Trial registration: to be completed |
| Objectives of trial, including interventions being investigated | | The primary objective of the CAPPT trial is to assess if providing a tailored dosage of oral iron-folic acid (IFA) supplementation along with a personalized package of nutrition education and counselling (the home visiting intervention) supported by community-based participatory learning and action (PLA) women's groups increases haemoglobin (Hb) levels at 28 weeks of pregnancy, compared with haemoglobin levels in women who have PLA women's groups only in their communities (PLA plus routine care) and with women who have access to routine antenatal care only (control).  Secondary objectives are as follows:  1. Identify whether a tailored home-visiting intervention supported by PLA women's groups and/or PLA women’s groups alone improve the following by 28 weeks gestation: Pregnant Women’s (PWs) dietary diversity and average daily intake of key micronutrients; equity of food and nutrient allocation between PW and their husbands; and compliance with recommended intake of prescribed supplements  2. Assess if any increases in haemoglobin (Hb) and secondary outcomes at 28 weeks are sustained or improved by 34 weeks gestation.  3. Identify the reported symptoms/side effects of iron supplements in groups according to dosage.  4. Evaluate who benefits most from the intervention.  6. Conduct a process evaluation to explore the mechanisms of effect and describe the context and implementation of the intervention  7. Conduct an economic evaluation to assess if the intervention is cost-effective, and what it costs to deliver at scale. |
| Outline of scope of Charter | | The purpose of this document is to describe the membership, terms of reference, roles, responsibilities, authority, decision-making and relationships of the Trial Steering Committee (TSC) for this trial, including the timing of meetings, methods of providing information to and from the TSC, frequency and format of meetings and relationships with other trial committees. |
|  | |  |
|  | |  |
| 2. Roles and responsibilities | |  |
| A broad statement of the aims of the TSC | | To act as the oversight body for this trial on behalf of the Sponsor/Funder. |
| Terms of reference | | The role of the TSC is to provide oversight for the trial. It should also provide advice through its independent Chair to the Trial Management Group (TMG), MRC, any other Funder on all aspects of the trial. |
| Specific roles of TSC | | - provide expert oversight of the trial - maintain confidentiality of all trial information that is not already in the public domain - make decisions as to the future continuation (or otherwise) of the trial/s - monitor recruitment rates and encourage the TMG to develop strategies to deal with any recruitment problems - approve the protocol(s) - review regular reports of the trial from the Trial Management Group (TMG) - receive letters of feedback from the DMC and consider their recommendations - assess the impact and relevance of any accumulating external evidence - monitor follow-up rates and review strategies from TMG to deal with problems - censure sites that are deviating from the protocol - approve any amendments to the protocol, where appropriate - approve any proposals by the TMG concerning any change to the design of the trial, including additional sub studies - oversee the timely reporting of trial results - approve / comment on the statistical analysis plan - approve external or early internal requests for release of data or subsets of data or samples including clinical data and stored biological samples |
| 3. Before or early in the trial | |  |
| Whether the TSC will have input into the protocol | | All potential independent TSC members should have opportunity to comment on the protocol as early as possible. Before recruitment begins the trial will have undergone review by the Sponsor/Funder (e.g. peer review for public sector trials), scrutiny by other trial committees and a research ethics committee. Therefore, if a potential independent TSC member has major reservations about the trial (e.g. the protocol, the logistics, ethical concerns) they should report these to the Chief Investigator and may decide not to accept the invitation to join. TSC members should be constructively critical of the ongoing trial, but also supportive of aims and methods of the trial. |
|  | |  |
| **Trial specific issues** | |  |
| Any issues specific to the disease under study | | Anaemia is a multi factorial problem and this trial is not designed to ameliorate anaemia other than that caused by iron deficiency and other micronutrients.  This is a pragmatic trial and will use existing stock of iron/folate tablets in each country. There is a possibility of low supply. |
| Any specific regulatory issues | | Consent and ascent issues have been discussed in detail with local ethics committees in each country. In Nepal, we will be recruiting women from the age of 13 and this has been approached with particular sensitivity and advice from the Nepal Research Ethics Committee. |
| Any other issues specific to the treatment under study | | None immediately apparent |
| Whether members of the TSC will have a contract | | TSC members will not be asked to formally sign a contract but should formally register their agreement to join the group by confirming (1) that they agree to be a member of the TSC and (2) that they agree with the contents of this Charter. Any potential competing interests should be declared at the same time. Members should complete and return the form in Annexes 1 or 2. Any observers (attendees who are not members) will sign a confidentiality agreement on the first occasion they attend a meeting (Annexe 3). |
| 4. Composition | |  |
| Membership and size of the TSC | | The majority of members of the TSC, including the Chair, should be independent ^[[1]](#footnote-1)^of the trial (see section 5). Non-independent members will also be part of the TSC.  The members of the TSC for this trial are:   1. *Professor Peter Brocklehurst – Independent member and chair of the Trial Steering Committee* 2. *Dr Kevin Kao – Independent member haematology (red cell) expert* 3. *Dr Shalini Singh – Independent member maternal health expert* 4. *Dr Madhu Devkota – Independent member – nutrition expert* 5. *Dr Umesh Kapil-Expert member from India – nutrition specialist* 6. *Dr Sara Hillman – Chief Investigator* |
| The Chair, how they are chosen and the Chair’s role. | | The Chair should have previous experience of serving on trial committees and experience of Chairing meetings, and should be able to facilitate and summarise discussions; knowledge of the disease area would be beneficial |
|  | |  |
| The responsibilities of the CI and other members of the TMG | | The CI (and, if appropriate, other TMG members) is an important member of the TSC and no major decisions should be made without their involvement. |
| The responsibilities of the observers | | Additional observers may be in attendance through (parts of) the TSC meetings in order to provide input on behalf of the trial’s Sponsor/Funder or to provide specific relevant expertise. |
| 5. Relationships | |  |
| Relationships with Chief Investigators, other trial committees (e.g. TMG and DMC), Sponsor/Funder and regulatory bodies | | The responsibilities of each trial committee are detailed in the protocol and in the respective Charters. |
| Advisory and executive bodies | The TSC is the oversight body and is delegated the roles in Section 2 by the Sponsor. All substantial issues regarding the trial must go to the TSC for consideration. The IDMC is advisory to the TSC. | |
| Payments to TSC members | | Members will be reimbursed for reasonable travel costs in country to attend meetings in person. No other payments or rewards would be given to professional members. |
| The need for TSC members to disclose information about any real or potential competing interests | | Any competing interests, both real or potential, should be disclosed. These are not restricted to financial matters – involvement in other trials or intellectual investment could be relevant. Although members may well be able to act objectively despite such connections, complete disclosure enhances credibility (See Annex 1).  TSC members should not use any trial data to inform trading in pharmaceutical shares, and careful consideration should be given to trading in stock of companies with competing products. Changes in declarations of real or potential competing interests should be minuted at the start of each meeting. |
| 6. Organisation of meetings | |  |
| Expected frequency of TSC meetings | | The TSC will meet at least yearly. At the request of the TSC, interim meetings, in person or by teleconference, will be organised. Many/major trial issues may need to be dealt with between meetings, by phone or by email. TSC members should be prepared for such instances. |
|  | |  |
| Attendance of TSC members at meetings | | Effort will be made for all members to attend. The Chief Investigator will work for a date that enables this. It is accepted that the CI may not attend all meetings where major actions are not expected. Members who cannot attend in person should be encouraged to participate by teleconference. If, at short notice, any TSC members cannot attend then the TSC may still meet if at least two independent members, including the Chair (unless otherwise agreed), will be present as well as a representative of the trial team. If the TSC is considering a major action after such a meeting the TSC Chair should communicate with the absent members, including the CI, as soon after the meeting as possible to check they agree. If they do not, a further teleconference should be arranged with the full TSC. |
| How TSC meetings will be organised, especially regarding open and closed sessions, including who will be present in each session | Presence will be usually limited to the TSC members, observers from the Sponsor/Funder, trials unit and the Facilitator. Other attendees may be invited for all or part of the meeting by the TSC including the trial statistician. The observers are not members of the TSC but may be invited to provide expert input or to represent the funding bodies involved; other observers will be at the discretion of the TSC and the Chief Investigator but may include members of the TMG other than the CI. | |
| Can TSC members who cannot attend the meeting input | If the report is circulated before the meeting, TSC members who will not be able to attend the meeting may pass comments to the TSC Chair, for contact for consideration during the discussions. | |
| What happens to independent members who do not attend meetings | If an independent member does not attend a meeting or provide comments when requested between meetings, it should be ensured that the independent member is available for the next meeting. If an independent member does not attend the next meeting or provide comments when next requested, they should be asked if they wish to remain part of the TSC. If an independent member does not attend a third meeting, strong consideration should be given to replacing this member. | |
| 7. Trial documentation and procedures to ensure confidentiality and proper communication | | |
| Intended content of material to be considered during meetings | A short report will be prepared by members from each country team. This will report on accrual and any matters affecting the trial. Additionally, the material may include a report *from* the DMC, requests *from* the TMG or draft publications. No trial outcome measure data will be presented by arm unless explicitly authorised by the DMC (eg toxicity). Where relevant, accrual, compliance with follow-up and adherence to treatment may be presented by centre. | |
| Whether reports to the TSC be available before the meeting or only at/during the meeting | It is usually helpful for the TSC to receive the report at least 1 week and preferably at least 2 weeks before any meetings. Different procedures may apply to teleconference meetings. | |
| The people who will see the accumulating data and interim analysis by randomised group | The accumulating trial data by arm and interim analyses will be confidential. These will be viewed only by the DMC. The TSC will not be routinely privy to these interim reports. The DMC will make recommendations to the TSC based on the interim data. | |
| Responsibility for identifying and circulating external evidence (e.g. from other trials/ systematic reviews) | Identification and circulation of external evidence (e.g. from other trials/ systematic reviews) is not the responsibility of the TSC members; it is a responsibility of the TMG. However, the TSC should continue to be made aware of other data that may impact on a trial. | |
| To whom the TSC will communicate the decisions that are made | (See Section 9) | |
| What will happen to the papers after the meeting | TSC members would be expected to delete, destroy or store securely copies of the reports to and from the TSC, agenda and minutes, as well as copies of communications between meetings. All documentation should be considered confidential. The Chief Investigator will keep a central record of all minutes, reports and correspondence by the TSC. | |
| 8. Decision making |  | |
| What decisions will be open to the TSC | Based on recommendations from the DMC, possible decisions include:-   - No action needed, trial continues as planned - Early stopping due, for example, to clear benefit or harm of a treatment, futility or external evidence - Modifying target recruitment, or pre-analysis follow-up, based on any change to the assumptions underlying the original trial sample size calculation (but not on any emerging differences) - Stopping one or more arms of a multi-arm trial - Sanctioning and/or proposing protocol changes   Based on other factors, possible decisions include the decisions above and:   - Approving proposed protocol amendments or new trial sub-studies - Approving requests for early release of (subsets of) data - Approving external applications for the use of stored samples - Approving presentation of results during the trial or soon after closure - Approval of new centres or strategies to improve recruitment or follow-up | |
| The role of formal statistical methods | Formal statistical methods may have been considered by the DMC in making their recommendations to the TSC. These methods are usually used as guidelines rather than absolute rules. This is because they generally only consider one dimension of the trial. The DMC will record reasons for disregarding a stopping guideline in the notes of their meetings and may choose to also note this in their report to the TSC if necessary. | |
| How decisions or recommendations will be reached within the TSC | Every effort should be made to achieve consensus. The role of the Chair is to summarise discussions and encourage consensus; therefore, it is usually best for the Chair to give their own opinion last.  It is important that the implications (e.g. ethical, statistical, practical, financial) for the trial be considered before any decision is made. | |
| When the TSC is quorate for decision-making | At least two independent members of the TSC should be present including the Chair, plus a representative of the and, if major action is to be considered, the CI. | |
| 9. Reporting |  | |
| To whom will the TSC report their recommendations/decisions, and in what form | The TSC will report their decisions to the TMG who will be responsible for implementing any actions resulting. The TSC may also provide feedback to the IDMC and, where appropriate, to the Sponsor/Funder. Copies of communications will pass through the Chief Investigator. Communications will be expected to be received by parties within 28 working days. | |
| Whether minutes of the meeting be made and, if so, by whom and where they will be kept | Notes of key points and actions will be made by the Rapporteur for the trial. This will include details of whether potential competing interests have changed for any attendees since the previous meeting. The draft minutes will be initially circulated for comment to those TSC members who were present at the meeting. The TSC Chair will sign off the final version of minutes or notes. | |
| What will be done if there is disagreement between the TSC and other trial committees | The TSC is the oversight body for the trial. However, the TSC should have good reason before deciding not to accept requests from the TMG and recommendations from the DMC. If there are serious problems or concerns with the TSC decision following an DMC recommendation, a joint meeting of the TSC and DMC should be held. The information to be shown would depend upon the action proposed and each committees’ concerns. Depending on the reason for the disagreement confidential data and/or data by trial arm may have to be revealed to all or some of those attending such a meeting: this would be minimised where possible. The meeting would be Chaired by a senior member of the UCL CTU staff or an external expert who is not directly involved with the trial. | |
| 10. After the trial |  | |
| Publication of results | The TSC will oversee the timely analysis, writing up and publication of the main trial results. The independent members of the TSC will have the opportunity to read and comment on the proposed main publications of trial data prior to submission. This review may be concurrent to that of the trial investigators and IDMC. | |
| The information about the TSC that will be included in published trial reports | TSC members will be named and their affiliations listed in the main report, unless they explicitly request otherwise. | |
| Any constraints on TSC members divulging information about their deliberations after the trial has been published |  | |

**Abbreviations and glossary**

| CI | Chief Investigator |
| --- | --- |
| DMC | Data Monitoring Committee |
| ISRCTN | International standard randomised controlled trial number |
| MRC | Medical Research Council |
| PW | Pregnant Woman |
| TMG | Trial Management Group |
| TSC | Trial Steering Committee |
| UAR | Unexpected adverse reaction |

## Annexe 1: Agreement and competing interests form for independent members

# CAAPT Trial Steering Committee: Agreement to join the Trial Steering Committee as an independent member and disclosure of potential competing interests

Please complete the following document and return to the TSC Facilitator.

(please initial box to agree)

|  | I have read and understood the TSC Charter version V1, dated 23/12/2019 |
| --- | --- |
|  | I agree to join the Trial Steering Committee for this trial as an independent member |
|  | I agree to treat all sensitive trial data and discussions confidentially |

The avoidance of any perception that independent members of a TSC may be biased in some fashion is important for the credibility of the decisions made by the TSC and for the integrity of the trial.

Potential competing interests should be disclosed. In many cases simple disclosure up front should be sufficient. Otherwise, the (potential) independent TSC member should remove the conflict or stop participating in the TSC. **Table 1** lists potential competing interests.

|  | **No,** I have no potential competing interests to declare |
| --- | --- |
|  | **Yes,** I have potential competing interests to declare (please detail below) |

| Please provide details of any potential competing interests: | |
| --- | --- |
|  |  |
|  |  |
|  |  |

Name: ___________________________

Signed: __________________________ Date: ______________

##### **Table 1: Potential competing interests for independent members**

| - Stock ownership in any commercial companies involved |
| --- |
| - Stock transaction in any commercial company involved (if previously holding stock) |
| - Consulting arrangements with the Sponsor/Funder |
| - Ongoing advisory role to a company providing drugs to the trial |
| - Frequent speaking engagements on behalf of the intervention |
| - Career tied up in a product or technique assessed by trial |
| - Hands-on participation in the trial |
| - Involvement in the running of the trial |
| - Emotional involvement in the trial |
| - Intellectual conflict e.g. strong prior belief in the trial’s experimental arm |
| - Involvement in regulatory issues relevant to the trial procedures |
| - Investment (financial or intellectual) or career tied up in competing products |
| - Involvement in the writing up of the main trial results in the form of authorship |

**Note:** This TSC charter was developed using MRC CTU template TSC Charter version 1.02, 13-Mar-2006

## Annexe 2: Agreement and competing interests form for non-independent members

# CAAPT Trial Steering Committee: Agreement to join the Trial Steering Committee as an non-independent member and disclosure of potential competing interests

Please complete the following document and return to the Facilitator.

(please initial box to agree)

|  | I have read and understood the TSC Charter version V1, dated23/12/2019 |
| --- | --- |
|  | I agree to join the Trial Steering Committee for this trial as an non-independent member |
|  | I agree to treat all sensitive trial data and discussions confidentially |

The avoidance of any perception that members of a TSC may be biased in some undisclosed fashion is important for the credibility of the decisions made by the TSC and for the integrity of the trial.

Possible competing interests should be disclosed. In many cases simple disclosure up front should be sufficient. Otherwise, the (potential) independent TSC member should remove the conflict or stop participating in the TSC. **Table 1** lists potential competing interests.

|  | **No,** I have no competing interests to declare other than involvement in the trial |
| --- | --- |
|  | **Yes,** I have competing interests to declare (please detail below) |

| Please provide details of any competing interests: | |
| --- | --- |
|  |  |
|  |  |
|  |  |

Name: ___________________________

Signed: __________________________ Date: ______________

##### **Table 1: Potential competing interests for non-independent members**

| - Stock ownership in any commercial companies involved |
| --- |
| - Stock transaction in any commercial company involved (if previously holding stock) |
| - Consulting arrangements with the Sponsor/Funder |
| - Ongoing advisory role to a company providing drugs to the trial |
| - Frequent speaking engagements on behalf of the intervention |
| - Intellectual conflict e.g. strong prior belief in the trial’s experimental arm |
| - Involvement in regulatory issues relevant to the trial procedures |
| - Investment (financial or intellectual) in competing products |

**Note:** This TSC charter was developed using MRC CTU template TSC Charter version 1.02, 13-Mar-2006

## Annexe 3: Agreement and confidentiality agreement for observers

# CAPPT Trial Steering Committee: Agreement to attend the Trial Steering Committee and treat all information confidentially

Please complete the following document and return to the Facilitator.

(please initial box to agree)

|  | I have received a copy of the TSC Charter version 1, 23/12/19 |
| --- | --- |
|  | I agree to attend the Trial Steering Committee meeting on ___/____/______ |
|  | I agree to treat as confidential any sensitive information gained during this meeting unless explicitly permitted |

Name: ___________________________

Signed: __________________________ Date: ______________

**Note:** This TSC charter was developed using MRC CTU template TSC Charter version 1.02, 13-Mar-2006

## Annexe 4: Summarise changes from previous version

**Version 1.0**

This is version 1.0 of the TSC charter for this trial. There are no changes to be reported.

1. Independence is defined in Table 1 of Annexe 1 [↑](#footnote-ref-1)
