# Supplementary material for: Comprehensive Anaemia Programme and Personalized Therapies (CAPPT): protocol for a cluster-randomised controlled trial testing the effect women’s groups, home counselling and iron supplementation on haemoglobin in pregnancy in southern Nepal
Source: Trials. 2022 Mar 1;23:183. doi: 10.1186/s13063-022-06043-z (PMC8886560; doi:10.1186/s13063-022-06043-z)
Supplement: Supplementary file 2 — Additional file 2: Supplementary Annex 2. List of selected study clusters with population and expected pregnancies. [file 13063_2022_6043_MOESM2_ESM.docx]

**Supplementary Annex 2: List of selected study clusters with population and expected pregnancies**

| Cluster number | Name of old VDC | Cluster Code | old-ward 1 | old-ward 2 | cluster is 1 old-ward | Women who consented to eligibility check (n) | Households that consented to the census | Male pop’ (n) | Female pop’ (n) | Total pop’ (n) | MWRA (13-49 years) (n) | Eligible MWRA consenting to menstrual monitoring follow-up (n) | Exp. preg/m 2.52 pr 100 population (n) | Exp. preg/m if 50% are <20 weeks (n) | Exp. preg/ m if 33% are <20 weeks | Exp. preg/m after 20% LTFU if 50% are <20 weeks (n) | Exp. preg/m after 20% LTFU if 33% are <20 weeks (n) | Exp. preg. over 6 m | Exp. preg. over 6m if 50% are <20 weeks (n) | Exp. preg. over 6m if 33% are <20 weeks (n) | Exp. preg. over 6m after 20% LTFU if 50% are <20 weeks (n) | Exp. preg. over 6 m after 20% LTFU if 33% are <20 weeks (n) |
| --- | --- | --- | --- | --- | --- | --- | --- | --- | --- | --- | --- | --- | --- | --- | --- | --- | --- | --- | --- | --- | --- | --- |
| 1 | Hathihawa | Ha1 | 2 | 4 | 0 | 427 | 482 | 2165 | 2090 | 4255 | 750 | 408 | 10.7 | 5.4 | 3.2 | 4.3 | 2.6 | 64.3 | 32.2 | 21.4 | 25.7 | 17.2 |
| 3 | Bijuwa | Bij1 | 5 | 8 | 0 | 382 | 410 | 1838 | 1671 | 3509 | 696 | 331 | 8.8 | 4.4 | 2.7 | 3.5 | 2.1 | 53.1 | 26.5 | 17.7 | 21.2 | 14.1 |
| 4 | Baskhore | Ba1 | 7 | 9 | 0 | 376 | 402 | 1828 | 1762 | 3590 | 703 | 366 | 9.0 | 4.5 | 2.7 | 3.6 | 2.2 | 54.3 | 27.1 | 18.1 | 21.7 | 14.5 |
| 7 | Lawani | L1 | 3 | 7 | 0 | 243 | 267 | 1082 | 974 | 2056 | 407 | 228 | 5.2 | 2.6 | 1.6 | 2.1 | 1.2 | 31.1 | 15.5 | 10.4 | 12.4 | 8.3 |
| 8 | Patariya | Pat1 | 4 | 7 | 0 | 555 | 613 | 2492 | 2373 | 4865 | 928 | 480 | 12.3 | 6.1 | 3.7 | 4.9 | 2.9 | 73.6 | 36.8 | 24.5 | 29.4 | 19.6 |
| 9 | Bithuwa | Bit1 | 5 | 6 | 0 | 253 | 276 | 1452 | 1416 | 2868 | 565 | 225 | 7.2 | 3.6 | 2.2 | 2.9 | 1.7 | 43.4 | 21.7 | 14.5 | 17.3 | 11.6 |
| 11 | Abhirao | A1 | 8 | 9 | 0 | 285 | 324 | 1342 | 1294 | 2636 | 471 | 270 | 6.6 | 3.3 | 2.0 | 2.7 | 1.6 | 39.9 | 19.9 | 13.3 | 15.9 | 10.6 |
| 12 | Dumara | Du1 | 5 | 7 | 0 | 248 | 265 | 1288 | 1154 | 2442 | 441 | 213 | 6.2 | 3.1 | 1.8 | 2.5 | 1.5 | 36.9 | 18.5 | 12.3 | 14.8 | 9.8 |
| 14 | Dharmpaniya | Dha1 | 1 | 6 | 0 | 274 | 290 | 1301 | 1227 | 2528 | 516 | 245 | 6.4 | 3.2 | 1.9 | 2.5 | 1.5 | 38.2 | 19.1 | 12.7 | 15.3 | 10.2 |
| 17 | Pakadi | Pak1 | 2 | 4 | 0 | 291 | 316 | 1285 | 1292 | 2577 | 527 | 256 | 6.5 | 3.2 | 1.9 | 2.6 | 1.6 | 39.0 | 19.5 | 13.0 | 15.6 | 10.4 |
| 18 | Pakadi | Pak2 | 8 | 9 | 0 | 317 | 340 | 1659 | 1592 | 3251 | 577 | 295 | 8.2 | 4.1 | 2.5 | 3.3 | 2.0 | 49.2 | 24.6 | 16.4 | 19.7 | 13.1 |
| 19 | Pipara | Pip1 | 1 | 2 | 0 | 185 | 192 | 1416 | 1258 | 2674 | 442 | 173 | 6.7 | 3.4 | 2.0 | 2.7 | 1.6 | 40.4 | 20.2 | 13.5 | 16.2 | 10.8 |
| 21 | Rangapur | R1 | 3 | 5 | 0 | 234 | 248 | 1159 | 1114 | 2273 | 461 | 192 | 5.7 | 2.9 | 1.7 | 2.3 | 1.4 | 34.4 | 17.2 | 11.5 | 13.7 | 9.2 |
| 23 | Parsohiya | Par1 | 7 | 8 | 0 | 241 | 256 | 1560 | 1395 | 2955 | 600 | 210 | 7.4 | 3.7 | 2.2 | 3.0 | 1.8 | 44.7 | 22.3 | 14.9 | 17.9 | 11.9 |
| 24 | Gauri | Gau1 | 1 | 3 | 0 | 226 | 232 | 1348 | 1275 | 2623 | 500 | 208 | 6.6 | 3.3 | 2.0 | 2.6 | 1.6 | 39.7 | 19.8 | 13.2 | 15.9 | 10.6 |
| 25 | Gauri | Gau2 | 8 | 9 | 0 | 215 | 231 | 1198 | 1042 | 2240 | 452 | 200 | 5.6 | 2.8 | 1.7 | 2.3 | 1.4 | 33.9 | 16.9 | 11.3 | 13.5 | 9.0 |
| 26 | Harnampur | Harn1 | 3 | 7 | 0 | 168 | 173 | 1066 | 927 | 1993 | 385 | 145 | 5.0 | 2.5 | 1.5 | 2.0 | 1.2 | 30.1 | 15.1 | 10.0 | 12.1 | 8.0 |
| 27 | Baluhawa | Balu1 | 2 | 3 | 0 | 352 | 363 | 1851 | 1731 | 3582 | 726 | 317 | 9.0 | 4.5 | 2.7 | 3.6 | 2.2 | 54.2 | 27.1 | 18.1 | 21.7 | 14.4 |
| 28 | Baidauli | Bai1 | 5 | 7 | 0 | 225 | 238 | 1157 | 1212 | 2369 | 448 | 181 | 6.0 | 3.0 | 1.8 | 2.4 | 1.4 | 35.8 | 17.9 | 11.9 | 14.3 | 9.6 |
| 29 | Somdiha | Som1 | 1 | 9 | 0 | 169 | 178 | 1016 | 886 | 1902 | 349 | 145 | 4.8 | 2.4 | 1.4 | 1.9 | 1.2 | 28.8 | 14.4 | 9.6 | 11.5 | 7.7 |
| 30 | Somdiha | Som2 | 5 | 6 | 0 | 210 | 220 | 989 | 992 | 1981 | 374 | 198 | 5.0 | 2.5 | 1.5 | 2.0 | 1.2 | 30.0 | 15.0 | 10.0 | 12.0 | 8.0 |
| 32 | Kajarhawa | Kaj1 | 1 | 2 | 0 | 188 | 205 | 1102 | 1080 | 2182 | 381 | 176 | 5.5 | 2.7 | 1.6 | 2.2 | 1.3 | 33.0 | 16.5 | 11.0 | 13.2 | 8.8 |
| 33 | Sauraha | Sau1 | 4 | 9 | 0 | 220 | 227 | 920 | 827 | 1747 | 371 | 201 | 4.4 | 2.2 | 1.3 | 1.8 | 1.1 | 26.4 | 13.2 | 8.8 | 10.6 | 7.0 |
| 34 | Sihokhore | Si1 | 4 | 5 | 0 | 209 | 221 | 1053 | 1020 | 2073 | 375 | 200 | 5.2 | 2.6 | 1.6 | 2.1 | 1.3 | 31.3 | 15.7 | 10.4 | 12.5 | 8.4 |
| 35 | Gotihawa | Go1 | 7 | 8 | 0 | 257 | 268 | 1187 | 1099 | 2286 | 444 | 238 | 5.8 | 2.9 | 1.7 | 2.3 | 1.4 | 34.6 | 17.3 | 11.5 | 13.8 | 9.2 |
| 36 | Sisawa | S1 | 2 | 3 | 0 | 298 | 321 | 1902 | 1691 | 3593 | 619 | 285 | 9.1 | 4.5 | 2.7 | 3.6 | 2.2 | 54.3 | 27.2 | 18.1 | 21.7 | 14.5 |
| 37 | Kushhawa | Ku1 | 4 | 6 | 0 | 199 | 215 | 1358 | 1202 | 2560 | 448 | 176 | 6.5 | 3.2 | 1.9 | 2.6 | 1.5 | 38.7 | 19.4 | 12.9 | 15.5 | 10.3 |
| 38 | Kushhawa | Ku2 | 8 | 9 | 0 | 238 | 262 | 1456 | 1321 | 2777 | 485 | 218 | 7.0 | 3.5 | 2.1 | 2.8 | 1.7 | 42.0 | 21.0 | 14.0 | 16.8 | 11.2 |
| 39 | Ajigara | Aj2 | 3 | 4 | 0 | 220 | 240 | 1089 | 991 | 2080 | 378 | 203 | 5.2 | 2.6 | 1.6 | 2.1 | 1.3 | 31.4 | 15.7 | 10.5 | 12.6 | 8.4 |
| 40 | Ajigara | Aj1 | 7 | 8 | 0 | 196 | 214 | 1087 | 1055 | 2142 | 361 | 178 | 5.4 | 2.7 | 1.6 | 2.2 | 1.3 | 32.4 | 16.2 | 10.8 | 13.0 | 8.6 |
| 41 | Bhalwari | Bha1 | 1 | 7 | 0 | 259 | 279 | 1369 | 1244 | 2613 | 497 | 227 | 6.6 | 3.3 | 2.0 | 2.6 | 1.6 | 39.5 | 19.8 | 13.2 | 15.8 | 10.5 |
| 44 | Maharajganj | M3 | 5 | 5 | 1 | 271 | 291 | 1598 | 1441 | 3039 | 483 | 250 | 7.7 | 3.8 | 2.3 | 3.1 | 1.8 | 45.9 | 23.0 | 15.3 | 18.4 | 12.3 |
| 45 | Bhilmi | Bhi1 | 3 | 9 | 0 | 251 | 276 | 1441 | 1328 | 2769 | 541 | 231 | 7.0 | 3.5 | 2.1 | 2.8 | 1.7 | 41.9 | 20.9 | 14.0 | 16.7 | 11.2 |
| 46 | Shivanagar | Shiv1 | 5 | 7 | 0 | 191 | 221 | 833 | 754 | 1587 | 263 | 177 | 4.0 | 2.0 | 1.2 | 1.6 | 1.0 | 24.0 | 12.0 | 8.0 | 9.6 | 6.4 |
| 47 | Shivanagar | Shiv2 | 8 | 9 | 0 | 291 | 337 | 1224 | 1083 | 2307 | 431 | 257 | 5.8 | 2.9 | 1.7 | 2.3 | 1.4 | 34.9 | 17.4 | 11.6 | 14.0 | 9.3 |
| 49 | Krishnanagar | K1 | 7 | 8 | 0 | 318 | 355 | 1470 | 1400 | 2870 | 490 | 281 | 7.2 | 3.6 | 2.2 | 2.9 | 1.7 | 43.4 | 21.7 | 14.5 | 17.4 | 11.6 |
| 51 | Bahadurganj | B2 | 3 | 3 | 1 | 243 | 266 | 1119 | 1054 | 2173 | 404 | 208 | 5.5 | 2.7 | 1.6 | 2.2 | 1.3 | 32.9 | 16.4 | 11.0 | 13.1 | 8.8 |
| 52 | Bahadurganj | B1 | 7 | 7 | 1 | 377 | 396 | 2126 | 1974 | 4100 | 776 | 297 | 10.3 | 5.2 | 3.1 | 4.1 | 2.5 | 62.0 | 31.0 | 20.7 | 24.8 | 16.5 |
| 53 | Sirsihawa | Sir1 | 8 | 9 | 0 | 165 | 195 | 921 | 817 | 1738 | 293 | 158 | 4.4 | 2.2 | 1.3 | 1.8 | 1.1 | 26.3 | 13.1 | 8.8 | 10.5 | 7.0 |
| 54 | Bhagwanpur | Bh1 | 1 | 2 | 0 | 176 | 196 | 1055 | 993 | 2048 | 328 | 170 | 5.2 | 2.6 | 1.5 | 2.1 | 1.2 | 31.0 | 15.5 | 10.3 | 12.4 | 8.3 |
| 55 | Bhagwanpur | Bh2 | 7 | 8 | 0 | 244 | 266 | 1780 | 1623 | 3403 | 547 | 226 | 8.6 | 4.3 | 2.6 | 3.4 | 2.1 | 51.5 | 25.7 | 17.2 | 20.6 | 13.7 |
| 56 | Ganeshpur | G1 | 1 | 4 | 0 | 204 | 242 | 881 | 858 | 1739 | 282 | 196 | 4.4 | 2.2 | 1.3 | 1.8 | 1.1 | 26.3 | 13.1 | 8.8 | 10.5 | 7.0 |
| 57 | Ganeshpur | G2 | 2 | 3 | 0 | 184 | 208 | 936 | 873 | 1809 | 299 | 170 | 4.6 | 2.3 | 1.4 | 1.8 | 1.1 | 27.4 | 13.7 | 9.1 | 10.9 | 7.3 |
| 58 | Pathardaiya | P2 | 5 | 5 | 1 | 286 | 332 | 1538 | 1558 | 3096 | 535 | 282 | 7.8 | 3.9 | 2.3 | 3.1 | 1.9 | 46.8 | 23.4 | 15.6 | 18.7 | 12.5 |
| 59 | Khurhuriya | Kh2 | 6 | 8 | 0 | 332 | 405 | 1535 | 1513 | 3048 | 524 | 316 | 7.7 | 3.8 | 2.3 | 3.1 | 1.8 | 46.1 | 23.0 | 15.4 | 18.4 | 12.3 |
| 62 | Bishunpur | Bi1 | 1 | 2 | 0 | 312 | 338 | 1392 | 1329 | 2721 | 486 | 308 | 6.9 | 3.4 | 2.1 | 2.7 | 1.6 | 41.1 | 20.6 | 13.7 | 16.5 | 11.0 |
| 66 | Manpur | Man1 | 7 | 8 | 0 | 235 | 267 | 1157 | 1078 | 2235 | 456 | 226 | 5.6 | 2.8 | 1.7 | 2.3 | 1.4 | 33.8 | 16.9 | 11.3 | 13.5 | 9.0 |
| 67 | Mahuwa | Ma1 | 1 | 3 | 0 | 297 | 341 | 1409 | 1293 | 2702 | 500 | 265 | 6.8 | 3.4 | 2.0 | 2.7 | 1.6 | 40.9 | 20.4 | 13.6 | 16.3 | 10.9 |
| 68 | Mahuwa | Ma2 | 5 | 6 | 0 | 256 | 290 | 1186 | 1157 | 2343 | 396 | 246 | 5.9 | 3.0 | 1.8 | 2.4 | 1.4 | 35.4 | 17.7 | 11.8 | 14.2 | 9.4 |
| 69 | Balaramwapur | Bal1 | 2 | 3 | 0 | 228 | 249 | 1149 | 1063 | 2212 | 414 | 223 | 5.6 | 2.8 | 1.7 | 2.2 | 1.3 | 33.4 | 16.7 | 11.1 | 13.4 | 8.9 |
| 70 | Dhankauli | D1 | 6 | 7 | 0 | 314 | 330 | 1520 | 1398 | 2918 | 611 | 250 | 7.4 | 3.7 | 2.2 | 2.9 | 1.8 | 44.1 | 22.1 | 14.7 | 17.6 | 11.8 |
| 72 | Niglihawa | N1 | 7 | 7 | 1 | 220 | 236 | 980 | 896 | 1876 | 392 | 178 | 4.7 | 2.4 | 1.4 | 1.9 | 1.1 | 28.4 | 14.2 | 9.5 | 11.3 | 7.6 |
| 76 | Jahadi | J1 | 2 | 9 | 0 | 131 | 172 | 639 | 660 | 1299 | 271 | 114 | 3.3 | 1.6 | 1.0 | 1.3 | 0.8 | 19.6 | 9.8 | 6.5 | 7.9 | 5.2 |
| 78 | Tilaurakot | Ti2 | 5 | 6 | 0 | 271 | 334 | 1121 | 1073 | 2194 | 401 | 231 | 5.5 | 2.8 | 1.7 | 2.2 | 1.3 | 33.2 | 16.6 | 11.1 | 13.3 | 8.8 |
|  | **totals** |  | **54** | **49** | **5** | **13957** | **15311** | **72025** | **67423** | **139448** | **25800** | **12648** | **351** | **176** | **105** | **141** | **84** | **2108** | **1054** | **703** | **843** | **562** |
|  | Total no of wards | |  | **103** |  |  |  |  |  |  |  |  |  |  |  |  |  |  |  |  |  |  |

Pop: population; MWRA: Married women of reproductive age; Exp. preg: Expected Pregnancies; LTFU: Loss to Follow-Up.
