# Supplementary material for: Comprehensive Anaemia Programme and Personalized Therapies (CAPPT): protocol for a cluster-randomised controlled trial testing the effect women’s groups, home counselling and iron supplementation on haemoglobin in pregnancy in southern Nepal
Source: Trials. 2022 Mar 1;23:183. doi: 10.1186/s13063-022-06043-z (PMC8886560; doi:10.1186/s13063-022-06043-z)
Supplement: Supplementary file 3 — Additional file 3: Supplementary Annex 3. Menstrual monitoring participant information sheet in English. [file 13063_2022_6043_MOESM3_ESM.docx]

**Supplementary Annex 3: Menstrual monitoring participant information sheet in English**

**Comprehensive Anaemia Program and Personalized Therapies (CAPPT) trial**

## Information sheet for menstrual monitoring

**Introduction**

Namaste! My name is ______________________. I have come from HERD International located in Thapathali, Kathmandu. HERD International is a national level research organization. This organization has been conducting various programmes and research in the health, environment, and social development. Currently, HERD International in partnership with University College London is conducting a study with an aim to reduce anaemia in pregnant women in Kapilbastu. I would like to invite you to be a part of this study.

Before you decide whether to participate, it is important for you to understand why this research is being done and what participation will involve. I will read what is written in this information sheet aloud to you. You can ask me if there is anything that you do not understand or if you want more information. You will be given a copy of this information sheet. Take your time to decide whether or not you want to take part in the study or not. Thank you for reading this/listening to me.

**Details of the study**

HERD International in partnership with University College London is conducting a study with an aim to reduce anaemia in pregnant women. The Medical Research Council (UK) is funding this research.

Anaemia is a condition when there is decreased haemoglobin in blood, and this is caused by various factors. In Nepal, lack of iron is the most common cause of anaemia in pregnancy. It is important to reduce anaemia in pregnancy because low iron levels are associated with illness and complications during pregnancy and childbirth. Pregnant women who are anaemic are much more likely to die during childbirth than those women who are not and their infants are more likely to be born small for gestational age.

In Nepal, Kapilbastu is one of the districts where anaemia is highly prevalent. Hence, we have chosen 54 areas (103 old wards) within 9 pallikas of Kapilbastu for this study. We are involving approximately 12700 pregnant women in Kapilbastu district in checking of their pregnancies and aim to enrol 1054 women. This research is designed to find out how anaemia in pregnant women can be reduced in this community and by doing the following:

1. Visiting the home of pregnant women by HERD staff to test their Haemoglobin levels and provide tailored iron-folic acid (IFA) tablets as per their anaemia status and nutrition counselling.
2. Mobilizing women’s groups to discuss anaemia, supplements, diet, and antenatal care in pregnancy using Participatory Learning and Action (PLA) method.

**Who are we inviting to participate?**

We need to enrol 1054 pregnant women who are early in their pregnancy in this study. For this, we need to monitor all the married women in your community to find out when they get pregnant. You can take part in this monitoring if you are a married woman or girl aged 13 to 49 years, living permanently in the study area, and could become pregnant (i.e., you and your husband have not had permanent contraception like laparoscopy, mini-laparoscopy or vasectomy or you have not attained menopause or not had a hysterectomy).

**What will happen if you agree to take part in checking of whether you are pregnant or not?**

If you agree, your area’s *Female Community Health Volunteer* will visit you at home and record information in a register book where we keep your name, address, and ID time. She will visit you every month for the next 7 months to check whether you might be pregnant by asking about your periods. If you have missed a period, she will ask you to give a urine sample to do a urine pregnancy test at your home which will help to confirm your pregnancy status. You do not have to pay money for doing the urine pregnancy test. The *Female Community Health Volunteer* will keep what you say confidential and won’t tell your family members or neighbours anything you share with her.

If you turn out to be pregnant and consent to participate in the study, the *Female Community Health Volunteer* will inform a HERD data collector who will visit you and give you a unique ID card and enrol you in the final study. Thereafter, HERD staff members will visit you a further four to six times to enquire about the progress of your pregnancy, diet, and ANC visits. We will measure your height and weight and do a finger prick to get a blood sample to know your haemoglobin level and anaemia status. We will tell you more about this in more detail if you become pregnant.

**Are there any risks if you participate?**

We do not think that any harm will come to you, but it is possible that you might find sharing information about your periods or pregnancy uncomfortable or upsetting. You don’t have to continue to take part if you don’t feel like it. If you would like to talk to someone about the feelings generated by the questions, please contact a member of HERD staff.

**Are there any benefits if you participate?**

If you agree to share your menstrual status each month with your *Female Community Health Volunteer*, we will offer to do a urine pregnancy test in your home after a missed period to confirm your pregnancy. You will not have to buy the urine pregnancy test kit nor pay anyone for doing your urine pregnancy test and you do not have to show the result to anyone except the FCHV. Knowing that you are pregnant at an early stage will help you plan your pregnancy, seek timely and appropriate health care for your unborn baby and eat nutritious food required for proper growth of your baby.

**Will my taking part in this project be kept confidential?**

All information you share with *Female Community Health Volunteer* or HERD staff will be kept strictly confidential, which means that they are not allowed to tell anyone what you have told them. The information will be recorded either on paper or entered onto mobile phones/ tablets.

In our records, a unique number will be used to identify you. Using this number instead of your name in our data will help to keep your identity anonymous to everyone except those researchers who are directly involving in coming to your house to talk to you. You will not be able to be identified in any ensuing reports or publications.

The kind of information we will keep about you will include your name, age or date of birth, address, and the answers to questions that we ask you or measurements we make on you. This information will be held securely either on paper and/or electronically at HERD International and in University College London in the UK under the provisions the local Data Protection laws. Your name will not be passed to anyone else outside the research team who is not involved in the trial or any future study to follow-up trial participants.

Your records will be available to people authorized to work on the trial and those responsible for ensuring that the study is carried out correctly. By signing the consent form, you agree to this access for the current study and any further research that may be conducted in relation to it, even if you withdraw from the current study.

If you withdraw consent from further study, unless you object, your data will remain on file and will be included in the final study analysis.

In line with Nepal and UK regulations, at the end of the study your data will be securely archived in UK and in Nepal.

**Ethical approval**

This study has been approved by the Nepal Health Research Council Approval ID number 353/2019, UCL Research Ethics Committee: Project ID number: 14301/001 and London School of Hygiene and Tropical Medicine ethics committee ID number: 16528.

**Agreeing to take part**

Your participation is voluntary. If you don’t want to take part, you can refuse without giving a reason. If you decide to take part in the menstrual monitoring, you will be given this information sheet to keep and be asked to sign or thumb print the consent form. If you agree to participate and then change your mind at any time, please tell us and we will stop visiting you. We will take a photo of the consent form with your signature which will be filed in your records. You can have more time to think this over if you are at all unsure.

**More information**

You should feel free to discuss the study with other people or ask us any questions.

If you have any more questions, you can contact, Trial Manager, HERD International, Prasuti Griha Marg, Thapathali, Kathmandu. Tel 01-4238045

or

Dr Naomi Saville, Senior Research Associate, University College London Institute for Global Health and Technical advisor to HERD, Kathmandu Nepal. Tel: 01-4238045

**HERD International District Office, Taulihawa, Kapilbastu. Tel: number: 076-590090**

**Thank you for reading this information sheet and for considering whether to take part in this research study.**
