# Supplementary material for: Comprehensive Anaemia Programme and Personalized Therapies (CAPPT): protocol for a cluster-randomised controlled trial testing the effect women’s groups, home counselling and iron supplementation on haemoglobin in pregnancy in southern Nepal
Source: Trials. 2022 Mar 1;23:183. doi: 10.1186/s13063-022-06043-z (PMC8886560; doi:10.1186/s13063-022-06043-z)
Supplement: Supplementary file 4 — Additional file 4: Supplementary Annex 4. Full Trial participant information sheets in English. [file 13063_2022_6043_MOESM4_ESM.docx]

**Supplementary Annex 4: Full Trial participant information sheets in English**

1. Control arm Participants

**Comprehensive Anaemia Program and Personalized Therapies (CAPPT) trial**

**Participant Information Sheet for Pregnant Women in Control Arm**

**Introduction**

Namaste! My name is ______________________. I have come from HERD International located in Thapathali, Kathmandu. HERD International is a national level research organization. This organization has been conducting various programmes and research in the health, environment, and social development. Currently, HERD International in partnership with University College London is conducting a study with an aim to reduce anaemia in pregnant women in Kapilbastu. I would like to invite you to be a part of this study.

Before you decide whether to participate, it is important for you to understand why this research is being done and what participation will involve. I will read what is written in this information sheet aloud to you. You can ask me if there is anything that you do not understand or if you want more information. You will be given a copy of this information sheet. Take your time to decide whether or not you want to take part in the study or not. Thank you for reading this/listening to me.

**Details of the study**

HERD International in partnership with University College London is conducting a study with an aim to reduce anaemia in pregnant women. The Medical Research Council (UK) is funding this research.

Anaemia is a condition when there is decreased haemoglobin in blood, and this is caused by various factors. In Nepal, lack of iron is the most common cause of anaemia in pregnancy. It is important to reduce anaemia in pregnancy because low iron levels are associated with illness and complications during pregnancy and childbirth. Pregnant women who are anaemic are much more likely to die during childbirth than those women who are not and their infants are more likely to be born small for gestational age.

In Nepal, Kapilbastu is one of the districts where anaemia is highly prevalent. Hence, we have chosen 54 clusters (103 old-wards) within 9 pallikas of Kapilbastu for this study. We are involving approximately 1054 pregnant women residing within these 54 clusters. These 54 clusters will be divided equally into 2 groups, each group comprising of 27 clusters and more than 500 pregnant women.

This research is designed to find out how anaemia in pregnant women can be reduced in this community by doing the following:

1. Visiting the home of pregnant women by HERD staff to test their Haemoglobin levels and provide tailored iron-folic acid (IFA) tablets as per their anaemia status and nutrition counselling.
2. Mobilizing women’s groups to discuss anaemia, supplements, diet, and antenatal care in pregnancy using Participatory Learning and Action (PLA) method.

**Who are we inviting to participate?**

You can take part in this research if you are a married woman or girl aged 13 to 49 years, pregnant at less than 20 weeks of gestation, planning to live in this study area for most of your pregnancy and are able to respond to the survey questions.

**What will happen if you agree to take part in this study?**

If you decide to take part and give your consent (by signing or thumb-printing a consent form), first I will give you a trial participant card with a unique identification number which will be your ID for this study period. I request you to keep this id card in a safe place so that when my other colleagues come to visit you, you can show them this card. You can also show this at the health facility to show the health worker that you are involved in the study. On a tablet, I will record some personal details about you such as your age, education, household details, medical and obstetric history. Also, today I will do a finger prick to obtain blood to test your Haemoglobin level using a Hemocue machine to check whether you have anaemia or not. I will also measure and record your weight, height, the circumference of your upper arm and ask you some questions about your eating habits, iron supplements, antenatal care, and any illness/discomfort you may have during pregnancy.

I will visit you again at around 30 weeks’ gestation and measure and record your Haemoglobin, weight, height, and the circumference of your upper arm. I will also enquire about your eating habits, iron supplements, antenatal care, and any illness/discomfort you may have during pregnancy.

Another HERD staff (dietary data assistant) may also visit you and your family at around 30 weeks’ gestation and again within the same week to ask you and your husband (or another male family member) in detail about what you ate the day before.

We will visit you again at 30 weeks of gestation and measure your haemoglobin level with a Hemocue machine (like we will do today) and record your Haemoglobin, weight, height, and the circumference of your upper arm. I will also enquire about your eating habits, iron supplements, antenatal care, and any illness/discomfort you may have during pregnancy. The dietary data assistant will also visit your twice around this time.

Your participation in this study will be complete after all three visits you to interview you have been completed.

**Are there any risks if you participate?**

Finger prick blood tests can be a little uncomfortable but there is minimal risk associated with them and we will follow all best practices of hygiene and infection control.

We do not think that any harm will come to you, but it is possible that you might find sharing information about your pregnancy uncomfortable or upsetting. You don’t have to continue to take part if you don’t feel like it. If you would like to talk to someone about the feelings generated by the questions, please contact a member of HERD staff.

**Are there any benefits if you participate?**

Participating in this study will help you know the level of haemoglobin in your body and find out whether you have anaemia or not. You will also know about your weight, height and mid arm circumference status which will help you understand about your nutritional health. Knowing that you are pregnant at an early stage will help you plan your pregnancy, seek timely and appropriate health care for your unborn baby and eat nutritious food required for proper growth of your baby.

At the end of your last interview, we undertake with you at around 30 weeks’ gestation you will receive Rs 1000 as a thank you for giving up your time to take part in the study.

**Will my taking part in this project be kept confidential?**

All the information that we collect about you during the course of the research will be kept strictly confidential. Only researchers directly associated with this project, who are involved in finding you to measure/interview you, will have access to your name and address. All other researchers who look at the information you share with us will not be able to identify you as your name and address will be removed. You will be allocated a trial participant card with a unique number, which will be used as a code to identify you instead of your name. You will not be able to be identified in any ensuing reports or publications.

If you consent to take part in this study, the records obtained while you are in this study (age, ethnicity, religion, education, haemoglobin concentration, measurements, symptoms of illness and so on) will remain strictly confidential at all times. The information will be held securely on either paper or electronically at HERD International and in University College London in the UK under the provisions the local Data Protection laws. Your name will not be passed to anyone else outside the research team who is not involved in the trial. Your records will be available to people authorised to work on the trial and those responsible for ensuring that the study is carried out correctly. By signing the consent form, you agree to this access for the current study and any further research that may be conducted in relation to it, even if you withdraw from the current study. Further research might involve other researchers using the information you give us, but without your name attached to it. Alternatively, we or other researchers might seek to find you in the future to undertake further research with you.

If you withdraw consent from further study, unless you object, your data and samples will remain on file and will be included in the final study analysis.

In line with Nepal and UK regulations, at the end of the study your data will be securely archived in UK and in Nepal.

**What will happen to the results of the research project?**

The results of the study will be available after it finishes and will be published in a scientific journal and presented at a scientific conference. The data will be anonymous and none of the participants in the trial will be identified in any report or publication. You will be able to see the study results (no personal information revealed) from a website that we will set up for this project.

**Data Protection Privacy Notice**

The data controller for this project will be University College London (UCL) and HERD International together. The UCL Data Protection Office provides oversight of UCL activities involving the processing of personal data and can be contacted at [data-protection@ucl.ac.uk](mailto:data-protection@ucl.ac.uk).

**Ethical approval**

This study has been approved by the Nepal Health Research Council Approval ID number 353/2019, UCL Research Ethics Committee: Project ID number: 14301/001 and London School of Hygiene and Tropical Medicine ethics committee ID number: 16528.

**Agreeing to take part**

Your participation is voluntary. If you don’t want to take part, you can refuse without giving a reason. If you decide to take part in the menstrual monitoring, you will be given this information sheet to keep and be asked to sign or thumb print the consent. If you agree to participate and then change your mind at any time, please tell us and we will stop visiting you. We will take a photo of the consent form with your signature which will be filed in your records. You can have more time to think this over if you are at all unsure.

**Contact for further information**

You are encouraged to ask any questions you wish, before, during or after getting involved. If you have any questions about the study, please speak to the HERD International researchers who visit you, who will be able to provide you with up-to-date information about the procedure(s) and supplements/ medicines involved. If you require any further information or have any concerns while taking part in the study, you can contact:

Trial Manager, HERD International, Prasuti Griha Marg, Thapathali, Kathmandu. Tel 01-4238045

or

Dr Naomi Saville, Senior Research Associate, University College London Institute for Global Health and Technical advisor to HERD, Kathmandu Nepal. Tel: 01-4238045

**HERD International District Office, Taulihawa, Kapilbastu. Tel: number: 076-590090**

**Thank you for reading this information sheet and for considering whether to take part in this research study.**

1. Intervention arm participants

**Comprehensive Anaemia Program and Personalized Therapies (CAPPT) trial**

**Participant Information Sheet for Pregnant Women Intervention Arm**

**Introduction**

Namaste! My name is ______________________. I have come from HERD International located in Thapathali, Kathmandu. HERD International is a national level research organization. This organization has been conducting various programmes and research in the health, environment, and social development. Currently, HERD International in partnership with University College London is conducting a study with an aim to reduce anaemia in pregnant women in Kapilbastu. I would like to invite you to be a part of this study.

Before you decide whether to participate, it is important for you to understand why this research is being done and what participation will involve. I will read what is written in this information sheet aloud to you. You can ask me if there is anything that you do not understand or if you want more information. You will be given a copy of this information sheet. Take your time to decide whether or not you want to take part in the study or not. Thank you for reading this/listening to me.

**Details of the study**

HERD International in partnership with University College London is conducting a study with an aim to reduce anaemia in pregnant women. The Medical Research Council (UK) is funding this research.

Anaemia is a condition when there is decreased haemoglobin in blood, and this is caused by various factors. In Nepal, lack of iron is the most common cause of anaemia in pregnancy. It is important to reduce anaemia in pregnancy because low iron levels are associated with illness and complications during pregnancy and childbirth. Pregnant women who are anaemic are much more likely to die during childbirth than those women who are not and their infants are more likely to be born small for gestational age.

In Nepal, Kapilbastu is one of the districts where anaemia is highly prevalent. Hence, we have chosen 54 clusters (103 old-wards) within 9 pallikas of Kapilbastu for this study. We are involving approximately 1054 pregnant women residing within these 54 clusters. These 54 clusters will be divided equally into 2 groups, each group comprising of 27 clusters and more than 500 pregnant women.

This research is designed to find out how anaemia in pregnant women can be reduced in this community by doing the following:

1. Visiting the home of pregnant women by HERD staff to test their Haemoglobin levels and provide tailored iron-folic acid (IFA) tablets as per their anaemia status and nutrition counselling.
2. Mobilizing women’s groups to discuss anaemia, supplements, diet, and antenatal care in pregnancy using Participatory Learning and Action (PLA) method.

**Who are we inviting to participate?**

You can take part in this research if you are a married woman or girl aged 13 to 49 years, pregnant at less than 20 weeks of gestation, planning to live in this study area for most of your pregnancy and are able to respond to the survey questions.

**What will happen if you agree to take part in this study?**

If you decide to take part and give your consent (by signing or thumb-printing a consent form), first I will give you a trial participant card with a unique identification number which will be your ID for this study period. I request you to keep this id card in a safe place so that when my other colleagues come to visit you, you can show them this card. You can also show this at the health facility to show the health worker that you are involved in the study. On a tablet, I will record some personal details about you such as your age, education, household details, medical and obstetric history. Also, today I will do a finger prick to obtain blood to test your Haemoglobin level using a Hemocue machine to check whether you have anaemia or not. I will also measure and record your weight, height, the circumference of your upper arm and ask you some questions about your eating habits, iron supplements, antenatal care, and any illness/discomfort you may have during pregnancy.

I will visit you again at around 30 weeks’ gestation and measure and record your Haemoglobin, weight, height, and the circumference of your upper arm. I will also enquire about your eating habits, iron supplements, antenatal care, and any illness/discomfort you may have during pregnancy.

Another HERD staff (dietary data assistant) may also visit you and your family at around 30 weeks’ gestation and again within the same week to ask you and your husband (or another male family member) in detail about what you ate the day before.

A female HERD staff who is an ANM (a “nutrition assistant”) will visit you in your home twice during your pregnancy, once at around 12-21 weeks’ gestation and another at 18-25 weeks’ gestation. At each visit she will discuss with you and your family members about your diet and health in pregnancy and give you advice based on what you are already eating / doing. She will check your anaemia (haemoglobin) level with a Hemocue machine (like we will do today) and tell you about your anaemia status and give you iron folic acid (IFA) tablets as per the standard treatment protocol followed in all government health facilities for treating anaemia in pregnancy.

The nutrition assistant will also invite you and your family members to attend monthly participatory women's groups (PLA meetings), which will be held in your community. You can attend these with a family member or a friend if you would like. The groups are open to anyone in the community. The nutrition assistant from HERD and your FCHV will facilitate these meetings and will discuss the problem of anaemia in pregnancy and how to reduce it through improved diet, eating iron-folic acid (IFA) tablets and going for antenatal care.

I will visit you again at 30 weeks of gestation and measure your haemoglobin level with a Hemocue machine (like we will do today) and record your Haemoglobin, weight, height, and the circumference of your upper arm. I will also enquire about your eating habits, iron supplements, antenatal care, and any illness/discomfort you may have during pregnancy. The dietary data assistant will also visit your twice around this time.

Your participation in this study will be complete after two visits from the nutrition assistant and three visits you to interview you have been completed.

**Are there any risks if you participate?**

Finger prick blood tests can be a little uncomfortable but there is minimal risk associated with them and we will follow all best practices of hygiene and infection control.

We do not think that any harm will come to you, but it is possible that you might find sharing information about your pregnancy uncomfortable or upsetting. You don’t have to continue to take part if you don’t feel like it. If you would like to talk to someone about the feelings generated by the questions, please contact a member of HERD staff.

**Are there any benefits if you participate?**

Participating in this study will help you know the level of haemoglobin in your body and find out whether you have anaemia or not. You will also know about your weight, height and mid arm circumference status which will help you understand about your nutritional health. Knowing that you are pregnant at an early stage will help you plan your pregnancy, seek timely and appropriate health care for your unborn baby and eat nutritious food required for proper growth of your baby.

You will receive two home visits from trained individuals who will provide you iron folic acid tablets as per your anaemia status and discuss with you and your family about nutrition in pregnancy to prevent anaemia. You and your family members may benefit from participating in the monthly women’s group meetings, which can be informative and enjoyable. If we find that you have severe anaemia, we will alert you to get medical assistance from the suitable health facility.

At the end of your last interview, we undertake with you at around 30 weeks’ gestation you will receive Rs 1000 as a thank you for giving up your time to take part in the study.

**Will my taking part in this project be kept confidential?**

All the information that we collect about you during the course of the research will be kept strictly confidential. Only researchers directly associated with this project, who are involved in finding you to measure/interview you, will have access to your name and address. All other researchers who look at the information you share with us will not be able to identify you as your name and address will be removed. You will be allocated a trial participant card with a unique number, which will be used as a code to identify you instead of your name. You will not be able to be identified in any ensuing reports or publications.

If you consent to take part in this study, the records obtained while you are in this study (age, ethnicity, religion, education, haemoglobin concentration, measurements, symptoms of illness and so on) will remain strictly confidential at all times. The information will be held securely on either paper or electronically at HERD International and in University College London in the UK under the provisions the local Data Protection laws. Your name will not be passed to anyone else outside the research team who is not involved in the trial. Your records will be available to people authorised to work on the trial and those responsible for ensuring that the study is carried out correctly. Also, your name and address will be shared with the health facility to inform them regarding your IFA intake from CAPPT project in order to prevent duplication of iron tablet consumption. By signing the consent form, you agree to this access for the current study and any further research that may be conducted in relation to it, even if you withdraw from the current study. Further research might involve other researchers using the information you give us, but without your name attached to it. Alternatively, we or other researchers might seek to find you in the future to undertake further research with you.

If you withdraw consent from further study, unless you object, your data and samples will remain on file and will be included in the final study analysis.

In line with Nepal and UK regulations, at the end of the study your data will be securely archived in UK and in Nepal.

**What will happen to the results of the research project?**

The results of the study will be available after it finishes and will be published in a scientific journal and presented at a scientific conference. The data will be anonymous and none of the participants in the trial will be identified in any report or publication. You will be able to see the study results (no personal information revealed) from a website that we will set up for this project.

**Data Protection Privacy Notice**

The data controller for this project will be University College London (UCL) and HERD International together. The UCL Data Protection Office provides oversight of UCL activities involving the processing of personal data and can be contacted at [data-protection@ucl.ac.uk](mailto:data-protection@ucl.ac.uk).

**Ethical approval**

This study has been approved by the Nepal Health Research Council Approval ID number 353/2019, UCL Research Ethics Committee: Project ID number: 14301/001 and London School of Hygiene and Tropical Medicine ethics committee ID number: 16528.

**Agreeing to take part**

Your participation is voluntary. If you don’t want to take part, you can refuse without giving a reason. If you decide to take part in the menstrual monitoring, you will be given this information sheet to keep and be asked to sign or thumb print the consent. If you agree to participate and then change your mind at any time, please tell us and we will stop visiting you. We will take a photo of the consent form with your signature which will be filed in your records. You can have more time to think this over if you are at all unsure.

**Contact for further information**

You are encouraged to ask any questions you wish, before, during or after getting involved. If you have any questions about the study, please speak to the HERD International researchers who visit you, who will be able to provide you with up-to-date information about the procedure(s) and supplements/ medicines involved. If you require any further information or have any concerns while taking part in the study, you can contact:

Trial Manager, HERD International, Prasuti Griha Marg, Thapathali, Kathmandu. Tel 01-4238045

or

Dr Naomi Saville, Senior Research Associate, University College London Institute for Global Health and Technical advisor to HERD, Kathmandu Nepal. Tel: 01-4238045

**HERD International District Office, Taulihawa, Kapilbastu. Tel: number: 076-590090**

**Thank you for reading this information sheet and for considering whether to take part in this research study.**
