# Supplementary material for: Comprehensive Anaemia Programme and Personalized Therapies (CAPPT): protocol for a cluster-randomised controlled trial testing the effect women’s groups, home counselling and iron supplementation on haemoglobin in pregnancy in southern Nepal
Source: Trials. 2022 Mar 1;23:183. doi: 10.1186/s13063-022-06043-z (PMC8886560; doi:10.1186/s13063-022-06043-z)
Supplement: Supplementary file 5 — Additional file 5: Supplementary Annex 5. Menstrual monitoring consent form in English. [file 13063_2022_6043_MOESM5_ESM.docx]

**Supplementary Annex 5: Menstrual monitoring consent form in English**

Patient Identification Number for this trial:

**CONSENT FORM FOR MENSTRUAL MONITORING**

**Title of Study: Comprehensive Anaemia Program and Personalized Therapies (CAPPT) trial**

Please complete this form after you have read the Information Sheet and/or listened to an explanation about the research.

Name and Contact Details of the Researcher(s):

**DR SUSHIL BARAL 9851068940 and DR NAOMI SAVILLE 9851017232**

This study has been approved by the Nepal Health Research Council Approval ID number 353/2019, UCL Research Ethics Committee: Project ID number: 14301/001 and London School of Hygiene and Tropical Medicine ethics committee ID number: 16528.

Thank you for considering taking part in this research. The researcher who has come to your home must explain the project to you before you agree to take part. If you have any questions arising from the Information Sheet or explanation already given to you, please ask the researcher before you decide whether to join in. You will be given a copy of this Consent Form to keep and refer to at any time.

Please complete this form after you have read the Information Sheet and/or listened to an explanation about the research. If you have any questions arising from the Information Sheet or explanation already given, please ask the researcher before you decide to participate in this study. You will be given a copy of this Consent Form to keep and refer to at any time.

I confirm that I understand that by ticking/initialling each box below I am consenting to this element of the study.

***In case of the married girl of age 14 to 19 running years (13 to 18 completed years), take the consent from her guardians as well. This means filling the tick box columns with the participant* and *her guardian and getting the signature or thumb print of both of them.***

|  | Woman’s Tick Box | Guardian’s Tick box |
| --- | --- | --- |
| I confirm that I have read the Information Sheet for the above study (or it has been read to me in an appropriate language) and I understood its content. |  |  |
| I have had an opportunity to consider the information and what will be expected of me and to ask questions which have been answered to my satisfaction. |  |  |
| I understand that my participation in this research is completely voluntary. I understand that I am free to withdraw from the research at any time without any repercussions. I understand that I can halt an interview at any point if I feel uncomfortable. |  |  |
| I understand the potential risks of participating and the support that will be available to me should I become distressed during the course of the research. |  |  |
| I understand that I will not benefit financially from this study or from any possible outcome it may result in in the future. |  |  |
| I understand that all personal information will remain confidential and that all efforts will be made to ensure I cannot be identified by anyone, except those who are involved in meeting me to take measurements and ask questions. |  |  |
| I understand that the data will not be made available to any commercial organisations but are solely the responsibility of the researcher(s) undertaking this study. |  |  |
| I understand that my data gathered in this study will be securely stored anonymously (without my name and address) and that it will not be possible to identify me in any publications. |  |  |
| I understand that my personal information and my data will not be possible to identify me in any publications. |  |  |
| I agree that my anonymised research data (that has my name and address removed) may be used by others for future research. I understand that no one will be able to identify you me if/when this these data are shared. |  |  |
| I would be happy for the data I provide, including my name and address, to be archived in the UCL Data Safe Haven to be kept there, and other secure locations in Nepal and UK, in case researchers need to find me again in the future. I understand that these personally identifiable data will not be available to anyone except authorised researchers who need my name and address to find me again in the future. |  |  |
| I understand that when I consent to have my photos taken while engaging in the research activities during the research period, that these could be published on HERD international’s or UCL’s website or other publications. |  |  |
| I would be happy to be contacted in future by HERD International and/or UCL researchers who would like to invite me to participate in follow-up studies to this project (for example if someone came to measure me or my child after this study ended) **.** |  |  |
| I have informed the researcher of any other research in which I am currently involved or have been involved in during the past 12 months. |  |  |
| **I consent to participate in this study** |  |  |

Signing this document means that you voluntarily agree to participate in this research after understanding the information provided to you.

| ***(In case the married participant’s age is between 14 and 19 running years (13 -18 completed years)***  ________________________ ________________ ___________________  Name of guardian Date Signature   \| Left \| Right \| \| --- \| --- \|   **OR Guardian’s**  **Fingerprints** |
| --- | --- | --- |

________________________ ________________ ___________________

Name of participant Date Signature

| Left | Right  Left |
| --- | --- |

**OR Participant’s**

**Fingerprints**

_____________________ ________________ ___________________

Researcher (Data Assistant) Date Signature
