# Supplementary material for: Comprehensive Anaemia Programme and Personalized Therapies (CAPPT): protocol for a cluster-randomised controlled trial testing the effect women’s groups, home counselling and iron supplementation on haemoglobin in pregnancy in southern Nepal
Source: Trials. 2022 Mar 1;23:183. doi: 10.1186/s13063-022-06043-z (PMC8886560; doi:10.1186/s13063-022-06043-z)
Supplement: Supplementary file 7 — Additional file 7: Supplementary Annex 7. Expected pregnancies to be enrolled and numbers after loss to follow-up. [file 13063_2022_6043_MOESM7_ESM.docx]

**Supplementary Annex 7. Expected pregnancies to be enrolled and numbers after loss to follow-up**

| **Number of pregnancies enrolled and measured in run-in period and full trial** | | | | | | | |
| --- | --- | --- | --- | --- | --- | --- | --- |
|  |  | **Before loss to follow-up** | | | **After loss to follow-up of 20%** | | |
| No of clusters per arm | % <20 weeks | Run-in period of 1 month | Full trial enrolment of 6 months | Total across the trial | Run-in period of 1 month | **Full trial enrolment of 6 months**^1^ | Total across the trial |
| Estimates per cluster | 50% | 3.3 | 19.5 | 22.8 | 2.6 | **15.6** | 18.2 |
|  | 33% | 2.2 | 13.0 | 15.2 | 1.7 | **10.4** | 12.1 |
| Estimates per arm | 50% | 88 | 527 | 614 | 70 | **421** | 491 |
|  | 33% | 59 | 351 | 410 | 47 | **281** | 328 |
| Estimates for whole trial | 50% | 176 | 1054 | 1229 | 140 | **842** | 983 |
|  | 33% | 117 | 702 | 819 | 94 | **562** | 655 |

^1^ number per cluster used in power calculation are given in this column. The power calculation assumes detectable difference of 0.4g/dl Hb, ICC (rho) 0.09, coefficient of variation of cluster size 0.27, 27 clusters per arm and SDs of 1.2 or 1.25
