# Supplementary material for: Comprehensive Anaemia Programme and Personalized Therapies (CAPPT): protocol for a cluster-randomised controlled trial testing the effect women’s groups, home counselling and iron supplementation on haemoglobin in pregnancy in southern Nepal
Source: Trials. 2022 Mar 1;23:183. doi: 10.1186/s13063-022-06043-z (PMC8886560; doi:10.1186/s13063-022-06043-z)
Supplement: Supplementary file 8 — Additional file 8: Supplementary Annex 8. Data Monitoring Committee (DMC) charter. [file 13063_2022_6043_MOESM8_ESM.docx]

**Supplementary Annex 8: Data Monitoring Committee (DMC) charter**

CAPPT Trial

Comprehensive Anaemia Programme and Personalized Therapies

**DATA MONITORING COMMITTEE CHARTER**

| **Study Title** | Cluster randomized controlled trial of a home counselling (and PLA in Nepal only) intervention to improve anaemia in pregnant women in rural India and Nepal |
| --- | --- |
| **Short Study Name** | CAPPT |
| **Trial registration** | ISRCTN registration number: 12272130 |
| **Ethical approval** | Ethical approval has been obtained from UCL ethics committee (Project ID number: 14301/001), Nepal Heath Research Council (NHRC approval number: 353/2019)  AIIMS Hospital Committee and London School of Hygiene and Tropical Medicine ethics committee (approval number: 16528). |
| **Sponsors** | AIIMS and HERD |
| **Version of the Document** | 2 |
| **Date of the current version** | 24 May 2021 |

**TABLE OF CONTENTS**

1. Trial overview 3

2. Outline and scope of DMC Charter 4

3. Role of DMC 4

4. Specific charges to the DMC in the CAPPT trial 4

5. DMC composition and individual responsibilities 5

6. Relationship with other committees 5

7. Frequency and format of meetings 6

8. Documentation and reporting 6

9. Blinding and confidentiality 6

10. Decision making 7

11. Post-trial interactions with the DMC 7

12. Expenses related to DMC attendance 7

**Appendix 1:** Possible competing interests to be disclosed prior to DMC meetings 7

**1. TRIAL OVERVIEW**

The aim is to improve maternal nutrition and prevent iron deficiency anaemia in pregnancy. The primary objective of the CAPPT trial is to assess, if providing a tailored dosage of oral iron- folic acid (IFA) supplements along with a personalized package of nutrition education and counselling (the home visiting intervention) supported by community-based participatory learning and action (PLA) women's groups, increases haemoglobin (Hb) levels at 30 weeks of pregnancy, compared with haemoglobin (Hb) levels in women who have access to routine antenatal care only (control).

In Nepal the trial will include two arms:

1. Control (routine care) arm where women have access to usual government services.

2) ’Home visiting’ intervention arm comprising of a home-based counselling with tailored iron/folic acid supplementation and participatory learning and action women's groups (PLA) held in the community for pregnant and non-pregnant women to attend in addition to routine care. During two home visits, the nutrition assistant tests the pregnant woman’s haemoglobin level and asks her about her diet and barriers to good nutrition. Then she provides a tailored dose of iron-folic acid tablets as well as counselling on how to improve diet.

In India no PLA will be offered and the study will comprise of two arms:

1. Control (routine care) arm

2) ’Home visiting ’ intervention arm comprising of a home-based counselling with tailored iron/folic acid supplementation with very similar content in both sites

The study is a non-blinded cluster-randomised controlled trial.

**Study partners:** The study activities are being led jointly by HERD in Nepal and All India Institute Medical Sciences (AIIMS, New Delhi. Funding for Nepal and UK budget through the MRC and in India from the Department of biotechnology (DBT).

**2. OUTLINE AND SCOPE OF DMC CHARTER**

The purpose of this document is to describe the roles and responsibilities of the independent DMC for the CAPPT trial, including the timing and format of meetings, methods of providing information to and from the DMC, and relationships with other committees. The Charter was prepared using the recommendations of the DAMOCLES group^[[1]](#footnote-1)^.

**3. ROLE OF DMC**

The role of the DMC is to:

- Protect and serve the CAPPT trial participants and to assist and advise the Principal Investigators so as to protect the validity and credibility of the trial.
- Safeguard the interests of the trial participants, assess the safety and efficacy of the interventions during the trial, and monitor the overall conduct of the trial.

The DMC will review the progress and data of this trial during an interim and final meeting and provide advice on its conduct to the Trial Steering Committee. After the final meeting, the DMC should inform the Chair of the Steering Committee if, in their view, the results are likely to convince a broad range of public health practitioners and researchers, that on balance one trial arm is clearly indicated or contraindicated, such that this evidence should inform current policy and practice.

**4. SPECIFIC CHARGES TO THE DMC IN THE CAPPT TRIAL**

The DMC will review the trial’s interim and final data, including figures on recruitment, data quality, and main outcome data. The charges to the DMC are to:

1. Monitor recruitment figures and loss to follow-up
2. Assess data quality, including completeness
3. Monitor compliance with the protocol by investigators
4. Monitor evidence for differences in the outcome measures, by arm
5. Decide whether to recommend that the trial should continue to recruit participants, or whether recruitment should be terminated early
6. Suggest additional data analyses if necessary
7. Advise on protocol modifications suggested by investigators or sponsors (e.g., recruitment length)
8. Monitor planned sample size assumption
9. Review the analysis plan for final trial analyses in due course
10. Monitor compliance with the interim DMC recommendations in the final DMC meeting

It is important for DMC members to read the trial protocol before participating in meetings. DMC members should be independent and constructively critical of the ongoing trial, but also supportive of its aims and methods. Any DMC members who wish to register a competing interest should inform one of the Principal Investigators (sara.hillman@ucl.ac.uk) prior to the first DMC meeting. Competing interests are not restricted to financial matters – involvement in other trials or intellectual investment are relevant. Although members will often be able to act objectively despite such connections, complete disclosure enhances credibility.

**5. DMC COMPOSITION AND INDIVIDUAL RESPONSIBILITIES**

The members of the DMC for the CAPPT trial are:

• Chair: Professor Keith West, Program Director, Human Nutrition Johns Hopkins University USA

• Mr James Martin, Institute of Applied Health Research, Research Fellow Institute of Applied Health Research, University of Birmingham, Birmingham, B15 2TT, UK [j.t.martin@bham.ac.uk](mailto:j.t.martin@bham.ac.uk)

• Dr. Rajendra Kumar BC -Research Advisor, Nepal Health Research Council, Kathmandu, Nepal. Research Team member of Nepal Micronutrient Status Survey (2016). Email: [drrajendra2005@gmail.com](mailto:drrajendra2005@gmail.com)

• Dr Meghnath Dhimal, Chief / Senior Research Officer, Health Research Section,

Nepal Health Research Council, Kathmandu, Nepal. Email: [meghdhimal@gmail.com](mailto:meghdhimal@gmail.com)

• Shobha Rao, Pune Maternal Nutrition Study

• Obstetric Haematologist- Dr Evangelia Koumoutsea Division of Hematology, Department of Medicine, Faculty of Medicine, University of Toronto. Email: evangelia.koumoutsea@uhn.ca

The investigators, trial advisory group and trial steering committee will be able to see data on the trial recruitment and follow-up combined across arms, data on the implementation of the intervention and data collection quality measures. They will not see the effect sizes for the primary outcome, which will only be seen by the DMC.

Team members from both sites will participate in the production of sections of the DMC report that present the trial recruitment and follow-up combined across arms, and the implementation of the intervention and data collection quality measures.

**6. RELATIONSHIP WITH OTHER COMMITTEES**

The CAPPT trial has two committees: a Trial Steering Committee (TSC) and a Data Monitoring Committee.

The role of the TSC is to:

1. Monitor and supervise the trial’s progress towards its interim and overall objectives
2. Advise the funders (Medical Research Council, and DBT) on the conduct and presentation of all aspects of the trial

The DMC recommendations should be circulated in writing to the TSC, with copies marked to the Trial Statistician (A Copas) and one of the Co-PIs (Naomi Saville) The recommendations will be discussed in a formal TSC meeting shortly after the DMC meeting. The DMC makes recommendations to the Chair of the TSC (Professor Peter Brocklehurst p.brocklehurst@bham.ac.uk), who is responsible for final executive decisions (e.g., about stopping or continuing the trial, or modifying the protocol).

**7. FREQUENCY AND FORMAT OF MEETINGS**

The DMC will meet twice during the course of the trial: in December 2019 and towards the end of the trial (final DMC meeting). Because the DMC includes India , Nepal, Canadian and UK-based members, we will seek to have as many members as possible in a face-to-face meeting in Delhi or Kathmandu, but some members may only be able to join by teleconference and this will be accommodated.

**8. DOCUMENTATION AND REPORTING**

Materials to be made available to the DMC prior to the meeting include:

- The DMC Charter
- The CAPPT trial protocol
- A data report, also containing a meeting-specific list of charges to the DMC

The DMC recommendations, signed and dated by the Chair, should be circulated to the Trial Steering Committee Chair (Dr Peter Brocklehurst p.brocklehurst@bham.ac.uk), the Trial Statistician ([a.copas@ucl.ac.uk](mailto:a.copas@ucl.ac.uk)) and one of the two co-PIs (Dr Sara Hillman; sara.hillman@ucl.ac.uk and Dr Naomi Saville; n.saville@ucl.ac.uk) within three weeks of the meeting.

**9. BLINDING AND CONFIDENTIALITY**

DMC members will not be blind to allocation. The DMC members should not circulate the confidential data report to anyone outside the DMC. DMC members should destroy their reports after each meeting. Fresh copies of previous reports will be circulated before each meeting.

**10. DECISION MAKING**

**Possible recommendations open to the DMC relating to each of the charges include:**

- Satisfactory progress, no action needed, continue as planned
- Early stopping due, for example to:

1. Serious problems in recruitment or retention.
2. Serious problems in implementing the intervention.
3. Serious error in the assumptions concerning the variability of the outcomes behind the sample size calculations (e.g., ICC).
4. A change in the environment of the trial which makes it no longer feasible or scientifically relevant (e.g., a change in government policy).
5. Another trial reports results that make the trial redundant.

- Extending recruitment and/or extending follow-up
- Sanctioning and/or proposing protocol changes

Decision making methods should be proposed by the DMC Chair and agreed upon by all members at the first DMC meeting. In general, it is recommended that every effort should be made for the DMC to reach a unanimous decision. If this cannot be achieved, a vote may be taken. The role of the Chair is to summarise discussions and encourage consensus; it may be best for the Chair to give their own opinion last.

There should be a minimum of three attendees before the DMC is quorate for decision making. Members who cannot attend in person should be encouraged to attend by teleconference. If, at short notice, any DMC members cannot attend at all then the DMC may still meet if at least one statistician and one clinician, including the Chair, will be present. If the DMC is considering recommending major action after such a meeting the DMC chair should talk with the absent members as soon as possible after the meeting to check that they agree. If they do not, a teleconference should be arranged with the full DMC.

As the data report will be circulated before the meeting, DMC members who cannot attend may pass comments to the DMC Chair for consideration during the discussions. If a member is not able to attend the interim meeting, they should be asked if they wish to remain a part of the DMC. If not, they should be replaced.

If the DMC has serious concerns with the TSC decision, a meeting of these two groups should be held.

**11. POST-TRIAL INTERACTIONS WITH THE DMC**

The final DMC meeting will be an opportunity for the members to discuss and give their advice about data interpretation to the investigators. DMC members will be named, and their affiliations listed in the main trial publication, unless they request otherwise. Details of the timings and deliberations of the DMC meeting may be included in this publication, if required (e.g., because of extensions to recruitment/follow-up, or changes to the protocol).

**12. EXPENSES RELATED TO DMC ATTENDANCE**

While the trial team does not offer honorariums for participating in DMC meetings, we will meet any in country travel or telephone expenses incurred by the members in relation to meetings.

**APPENDIX 1: Possible competing interests to be disclosed prior to DMC meetings**

The avoidance of any perception that members of a DMC may be biased in some fashion is important for the credibility of the decisions made by the Committee, and for the integrity of the trial as a whole. Possible competing interests should be disclosed to Dr Hillman and the Trial Statistician (Dr A Copas). In most cases, simple disclosure should be sufficient. Otherwise, the (potential) DMC member should remove the conflict or stop participating in the DMC. Below is a list of potential competing interests.

- Career tied up in a form of intervention assessed by the trial
- Hands-on participation in the trial
- Intellectual conflict, e.g., strong prior belief in or against the trial’s experimental intervention

1. DAMOCLES Study Group. A proposed charter for clinical trial data monitoring committees: helping them do to their job well. *Lancet* 2005; **365**: 711-22. [↑](#footnote-ref-1)
